# Contributing to a value-based health care framework for lung cancer patients in Switzerland – A methodological approach to merge routinely collected hospital data

**DOI:** 10.1371/journal.pone.0327814

**Published:** 2025-07-10

**Authors:** Michaela Carla Barbier, Katya Galactionova, Mark Lambiris, Leonel Oliveira, Florian Rüter, Dominik Glinz, Jessica Thürmer, Flurina Pletscher, Benjamin Kasenda, Tobias Finazzi, David König, Didier Lardinois, Larissa Conrad, Leonie Mutz, Matthias Schwenkglenks

**Affiliations:** 1 Institute of Pharmaceutical Medicine (ECPM), University of Basel, Basel, Switzerland; 2 Health Economics Facility, Department of Public Health, University of Basel, Basel, Switzerland; 3 Quality Management and Value-based Health Care, University Hospital Basel, Basel, Switzerland; 4 Roche Pharma (Schweiz) AG, Basel, Switzerland; 5 Medical Oncology, University and University Hospital Basel, Basel, Switzerland; 6 Clinic of Radiotherapy and Radiation Oncology, University Hospital Basel, Basel, Switzerland; 7 Department of Thoracic Surgery, University Hospital Basel, Basel, Switzerland; 8 Medical Controlling, Finance Department, University Hospital Basel, Basel, Switzerland; Universitas Pendidikan Ganesha, INDONESIA

## Abstract

**Background:**

The concept of Value-Based Health Care (VBHC) seeks to maximise patient value by optimising health outcomes considering costs. However, necessary data are not readily available. In Switzerland, hospital accounting is disconnected from patient outcomes and treatment data. We demonstrate the feasibility of merging routine hospital data, including patient-reported outcome measures (PROMs), to implement core elements of VBHC in a real-world lung cancer centre.

**Methods:**

We developed a merging approach using records from 208 newly diagnosed lung cancer patients treated at University Hospital Basel between June 2020 and November 2023. Maximum patient follow-up was 12-months. Clinician-reported outcome measure (CROM) and PROM data collection followed the International Consortium for Health Outcomes Measurement (Lung Cancer Set) standard. Cost data were extracted from Switzerland’s standard hospital accounting system (REKOLE^®^). To illustrate analytical options offered by the merged data, we analysed partial correlations between costs and utility changes from baseline.

**Results:**

The merging approach successfully allocated costs to specific lung cancer treatments and separated costs for comorbidity care, enabling an initial presentation of cost distributions for different elements of care. Median total first-year hospital costs per lung cancer patient were CHF 77,834 (mean CHF 93,621). Immunotherapy incurred the highest median costs of CHF 45,394 (mean CHF 49,518), followed by surgery of CHF 41,665 (mean CHF 48,940). First-year costs for patients diagnosed with stage I tended to be lower than for later stages. A standard graphical tool was developed to track individual patient treatment, outcome, and cost over time.

**Conclusions:**

This proof-of-concept analysis demonstrates the feasibility of a novel data merging approach as a foundation for VBHC implementation. While limited by sample size and follow-up duration, our method supports future treatment-cost-benefit models. It is reproducible and scalable across other conditions and hospitals, enabling the development of lung cancer treatments towards greater value and efficiency.

## Introduction

The transition towards the Value-Based Health Care (VBHC) concept is increasingly recognized as a critical strategy for improving patient outcomes while controlling healthcare costs [1–4]. Originally introduced by Porter and Teisberg, VBHC defines value in healthcare as the health outcomes achieved per dollar spent, thus shifting the focus from volume to value in care delivery [5–7].

VBHC expands on evidence-based medicine by incorporating shared decision-making and aims for a more patient-focused, value-driven treatment strategy across the full cycle of medical care [1]. All resource utilizations and related expenditures (including those for treatments, complications and comorbidities), as well as clinical and patient outcomes should be evaluated and optimised in such a way that the best possible quality of care and benefit are achieved for the patient while controlling cost [1,4,8].

Porter and Teisberg operationalized VBHC through six core components: (i) organize care into integrated practice units, (ii) measure outcomes and costs for every patient, (iii) move to bundled payments for care cycles, (iv) integrate care delivery systems, (v) expand geographic reach, and afterwards (vi) build an enabling information technology platform [6]. However, while their framework outlines the essential elements, it still offers only partial guidance on the practical implementation and implementation strategies suited for different healthcare contexts [9–11].

The second component of VBHC is often operationalised with disease-specific standardised patient-reported outcome measures (PROMs). These instruments collect information on outcomes that matter most to patients such as quality of life, recovery time, and satisfaction with care. PROMs are essential for patient empowerment and engagement, facilitating shared decision making in selecting appropriate care options [12]. This transcends the traditional approach where the sole decision-makers were clinicians who drew on treatment guidelines informed by clinical trial results in select patient populations. It is encouraging that a growing number of major Swiss healthcare institutions have integrated systematic PROM collection into strategic planning or are piloting PROM implementations [13].

Despite widespread consensus on the importance of VBHC among clinicians, hospital administrators, and patients, its implementation remains constrained, with the lack of integrated data systems being one major challenge. A key barrier in the Swiss context is the Swiss Federal Act on the Old-Age and Survivors’ Insurance (“AHVG”) [14], which limits the use of the AHV number to social insurance purposes in order to protect personal data. As a result, financial data that are routinely recorded for hospital billing and reimbursement are disconnected from both clinical and patient-reported outcome data. Methods like Time-Driven Activity-Based Costing [4] are useful tools for tracking the cost of patient care across the entire care cycle (requiring detailed time tracking and cost data), but are very resource-intensive and costly, and hence impractical for routine use in daily practice. Therefore, there is a pressing need for pragmatic approaches that leverage existing data.

**Fig 1:**
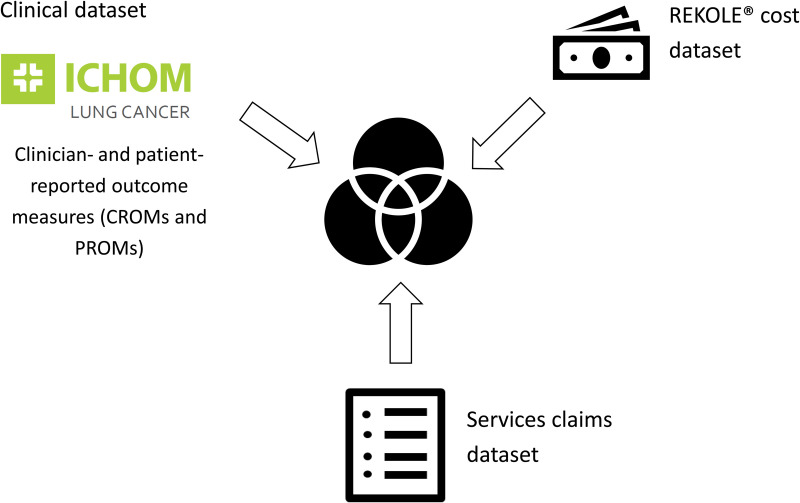
Merge of available hospital datasets. Abbreviations: ICHOM, International Consortium for Health Outcomes Measurement.

In Switzerland, three types of hospital datasets are routinely collected: a clinical dataset (including PROMs), as well as a service and a cost dataset (Fig 1). Although these datasets were not originally designed to be combined, we found that merging them is possible, albeit complex. We therefore propose a novel methodological approach for linking and mapping available patient data, using the University Hospital Basel (USB) lung cancer department as a pilot.

This article outlines the derived method and uses data accumulated over one year to generate exploratory proof-of-concept results. It suggests approaches to validation of the resulting estimates and highlights analytical techniques – including Spearman correlation and partial correlations within structural equation models (SEM) – that can be applied to the merged data to glean novel insights on the costs and benefits of different therapeutic options. Extended follow-up (FU) periods and ongoing patient recruitment will enable more sophisticated analyses of associations between lung cancer costs, individual patient treatments and benefits. Building on these preliminary findings, our study exemplifies the implementation of two key components of the VBHC framework: “measure outcomes and costs for every patient” and “build an enabling information technology platform.” We also describe the concrete strategy developed to operationalize these components in a real-world hospital lung cancer setting. By demonstrating how routinely collected hospital data can be systematically linked and utilized to inform value-based decisions, our approach offers a replicable model for other Swiss institutions or hospitals in other countries with similar data structures aiming to move from VBHC theory to practice.

## Materials and methods

### Ethics and data responsibilities

Clarification request Req-2021-01501 to the Ethics Committee Northwest and Central Switzerland (EKNZ) revealed that the project did not fall within the scope of the Human Research Act. Informed consent for data use was available from 284 patients. Data from further 9 patients could be used without informed consent (8 deceased, 1 not contactable) based on a second request (2023-01582) to the EKNZ which approved the research project as a further use of health-related personal data in the absence of consent (Art. 34 HRA, Art. 37-40 HRO).

The USB financial directorate was responsible for the preparation and monitoring of the raw (CROM, PROM, service, and cost) data analysed in this study. The Department of Clinical Pharmacology and Epidemiology (ECPM) at the University of Basel, which conducted the analysis, was responsible for the derived cost estimates.

### Population, study setting, and study perspective

We performed a descriptive observational study. We enrolled all consenting adult patients (written informed consent or ethical waiver 2023−01582) who were newly diagnosed with lung cancer at USB between June 2020 and June 2023. Eligibility conditions comprised small cell lung cancer, non-small cell lung cancer, lung cancer not otherwise specified, thoracic tumours, and others, irrespective of the stage of disease (S1 Fig in S1 Appendix, n = 293) [15]. Eligible patients were identified at weekly multidisciplinary tumour board meetings. Patients were followed up for a maximum of one year, or until the data cut-off point of this interim analysis, i.e., November 24, 2023 (S1 Section in S1 Appendix). The mean patient FU duration was 10 months for patients who were not deceased.

Some of the enrolled patients had their residency in the Swiss canton of Jura. USB maintains contractual agreements with the Delémont Cantonal Hospital allowing patients to obtain part of their medical treatment at USB. Since no information was available on the percentage amount of the services received at USB or at Delémont Cantonal Hospital, and since merging of incomplete datasets would have led to errors, we excluded all patients from the canton Jura from our full analysis set (FAS) population. One patient from the canton Zurich with incomplete data was additionally excluded. Patients simultaneously participating in a clinical trial were also excluded due to the unknown influence on our analyses results. Some services covered by clinical study budgets might not have shown up in the hospital accounting data.

We adopted the USB hospital perspective, i.e., only USB inpatient and USB outpatient medical services and costs were included in our analysis.

Throughout the duration of this project, the collaborators at USB Basel who had the appropriate access rights had access to individual patient data within the clinical information system. The clinical and financial data of enrolled patients was pseudonymised and extracted from their respective databases by the USB database owners. To ensure data security during transfer, only pseudonymised data records were transmitted from the University Hospital Basel to ECPM at the University of Basel via a password-protected Secure Transfer. For research purposes, the department ECPM of the University of Basel received at least 12 updates of the pseudonymised clinical and accounting data during the conduct of the project. The first “example data” delivery was accessed and sent to ECPM on Jan 14, 2022 and most recent data on March 12, 2024. Only dedicated research collaborators from ECPM University of Basel were allowed access to the research database.

### Description of the datasets

#### Clinical dataset.

All PROMs and clinician reported outcome measures (CROMs) were collected according to the lung cancer standard set developed by the International Consortium for Health Outcomes Measurement (ICHOM) [16] and manually entered in the Heartbeat® software [17]. The USB used the EORTC quality of life questionnaires QLQ-C30 and QLQ-LC29 to capture PROMs at baseline (BL), 3, 6, and 12 months after diagnosis [18]. Relevant CROM variables were retrospectively extracted from electronic health records.

CROMs consisted of case-mix variables, treatment variables, and outcomes. S1 Table in S1 Appendix presents a simplified excerpt from the original CROM/PROM dataset, and S2 Table in S1 Appendix a derived dataset. S2 Section in S1 Appendix describes the derived comorbidity score. CROM/PROM data stored in Heartbeat^®^ are from now on referred to as the clinical dataset.

#### Service dataset.

Financial reporting and billing data are highly harmonised across hospitals in Switzerland and allow the allocation of costs to specific patients, but they do not allow for a direct linkage with clinical data. For this reason, a third dataset covering all hospital medical service claims (henceforward: service dataset) was required to link and merge the clinical dataset and the cost dataset. In this and the next section, we elaborate on the structure of the service and the cost datasets.

The service dataset (tailored to the needs of hospital finance and accounting departments) collects individual hospital service claims by patient identification number and case number (S3 Table, S3 Section in S1 Appendix). Patients have one unique identification number and at least one, but usually several, case numbers assigned. One case number covers all individual service claims either related to an inpatient stay or to hospital outpatient care, the latter for a maximum period of up to 12 months (after the first outpatient visit for the same main diagnosis). The different case numbers per patient are created over the full medical care cycle. Inpatient and outpatient service claims are always stored under different case numbers. For a further inpatient stay of the same patient, the same case number would only be used if the patient was readmitted with the same main diagnostic category within 18 days after previous discharge. To detail the various service claims within a case number, additional variables are employed, denoting the type of service (e.g., physician visits, drug codes, imaging specifications, meals), case type (outpatient or inpatient care), and organisational units. Organisational units specify the hospital wards receiving or discharging the patient, or the wards in which medical services are delivered (called executing wards). However, the related overarching patient centred treatment (as collected in the clinical dataset: surgery, chemotherapy, radiotherapy, immunotherapy, and targeted therapy) is not directly stated in the service dataset or in the cost dataset.

#### Cost dataset.

For the financial reporting and billing of services, Swiss hospitals uniformly use the standardised operational accounting system Revision of Cost Accounting and Performance Recording^®^ (REKOLE^®^), which generates a separately managed cost dataset next to the clinical and the service datasets. A nationwide REKOLE^®^ seal of approval certifies high transparency and comparability with other hospitals [19].

A common variable in both the cost and the service dataset is the case number. However, the cost dataset does not contain the patient identification number. Variables in the cost dataset, but not included in the services dataset, are the “costs” (real costs incurred at the hospital), “month of billing”, “year of billing”, as well as different cost (centre) types (S4 Table in S1 Appendix). “Month” and “year” of billing represent the date a service is billed and not when the service is provided (the billing could be months after the actual date of the service provision). Therefore, these variables cannot be used for merging purposes.

Because the cost dataset does not contain the patient identification number or exact medical service dates, and because the clinical dataset does not contain case numbers, the service dataset was necessary to link the information per patient and per case number from all three datasets. It also offered the possibility to reduce the service and the cost dataset to case numbers related to services during our analysis period only. More details about the variables, dataset structure, data security, and resulting challenges are outlined in S5 and S6 Tables and S4 Section in S1 Appendix.

### Methods

#### Merge strategy step 1.

**Fig 2:**
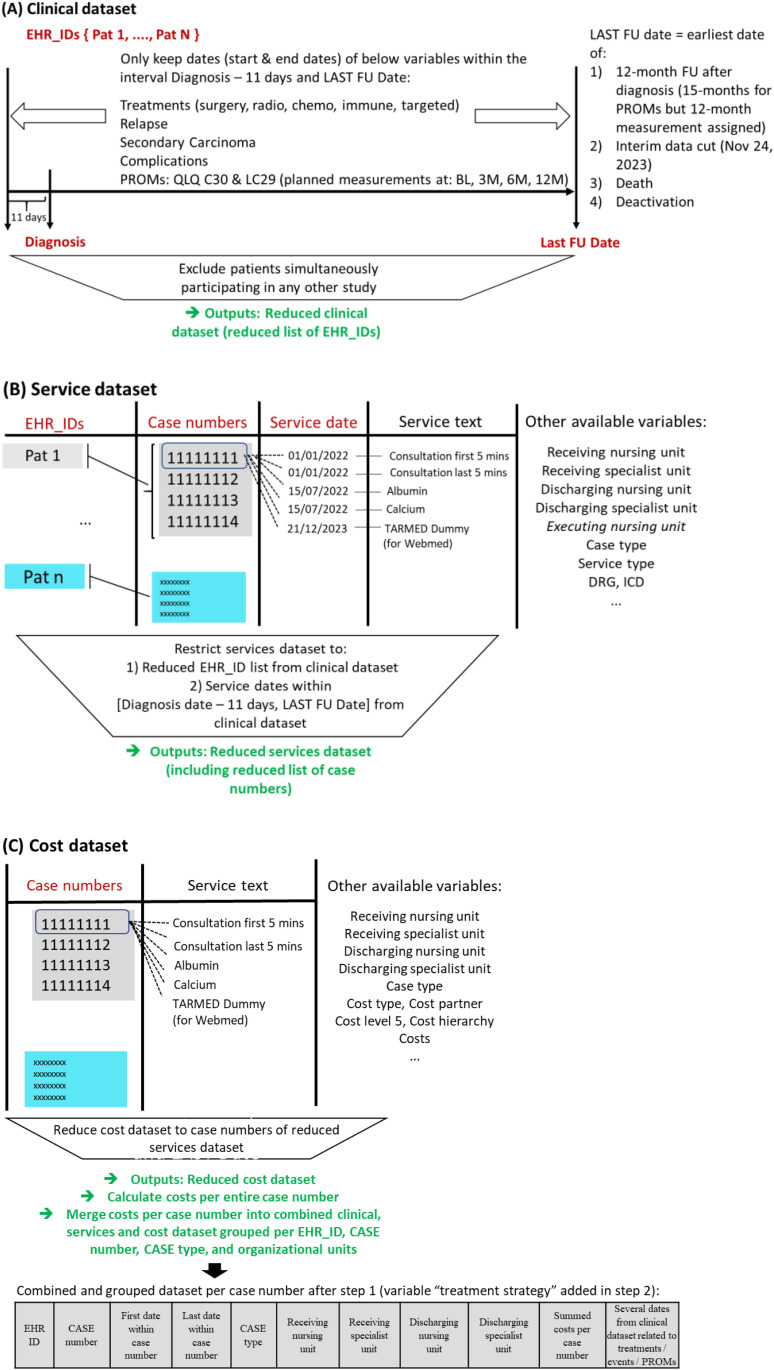
First part of the merge strategy for clinical dataset (A), service dataset (B), and cost dataset (C). Abbreviations: EHR electronic health record; FU follow-up; ID identifier; M month; pat patient; PROM patient-reported outcome measure; QLQ quality of life questionnaire.

We gradually merged and reduced the clinical, service and cost datasets, only retaining information for our eligible patients, FU period, and the variables outlined at the end of Fig 2 and S7 Table in S1 Appendix. Fig 2 describes the complete step 1 of the developed merging process, with S5 Section in S1 Appendix providing additional information. The content of our newly derived dataset is grouped by case number and is called “grouped dataset” from now on. S7 Table in S1 Appendix shows a snippet. It contains one line per case number, the first and last service date that was billed within the case number, the summed costs for the entire case number, and other variables that were available in both the service and the cost dataset (in particular, case type and organisational units). The grouped dataset does hence no longer contain all individual service claims. From the clinical dataset, important dates related to treatment start and end, complications, and PROM measurement time points were added per patient identification number.

#### Merge strategy step 2.

**Fig 3:**
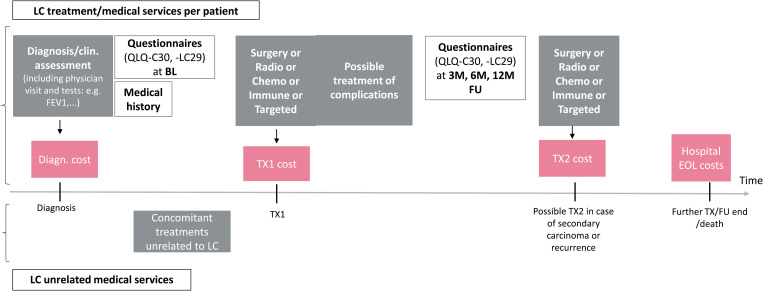
Costs for key medical services over time. Abbreviations: BL baseline; Diagn diagnosis; EOL end of life; FEV forced expiratory volume; FU follow-up; LC lung cancer; M month; QLQ quality of life questionnaire; TX treatment.

The grouped dataset prepared in step 1 does not directly reveal the underlying treatment(s) associated with a case number. While it provides all treatment and event dates (e.g., for complications) for each patient, the time span from the start to the end of a particular treatment (e.g., chemotherapy) may overlap partially or completely with the time spans of multiple case numbers. To create an additional variable, “treatment”, in the grouped dataset – representing the specific treatment or treatments related to a case number – we used treatment start and end dates (from the clinical dataset) and targeted search queries. The queries were derived after consultation with our medical experts and applied to the ungrouped service and cost datasets, which provided the necessary level of detail. The search queries used, among others, the variable “case type” (e.g., requirement of an inpatient stay for a surgery), specific organisational units (e.g., “thoracic surgery”), particular drug names (to identify different systemic treatments) and diagnosis-related group (DRG) codes. We searched for drug names and DRGs in the variable “service text”. The match of a specific drug name in the ungrouped datasets assigned the entire case number in the grouped data set to this treatment, provided no further match was found. The search queries are outlined in detail in S8 and S9 Tables (with further details given in S6 Section in S1 Appendix). We assumed that complications of lung cancer treatment would generally be billed within the same case number as the treatment that triggered them. Apart from treatments, we also assigned “diagnosis” and “death in hospital” to case numbers (Fig 3). “Diagnosis” could only be assigned if there was at least one unassigned case number before starting the first treatment. We also included a category labelled “Other” to represent unassigned case numbers and comorbidity treatment. S10 Table in S1 Appendix shows an outline of the grouped data after step 2. In case of combined treatment administration (e.g., chemo-radiotherapy), we represented the related case number by several lines (one for each treatment) and broke down the summed costs of the respective case. We categorised costs into (1) costs associated with identified drugs and (2) radiotherapy costs related to our specific radiotherapy search queries, where applicable. The remaining costs were equally split among the multiple treatments assigned (3).

#### Proof-of-concept of the developed merging approach and validation.

As a proof of concept, we applied our newly developed merging approach to the three previously described USB datasets. We assessed the quality of the merge by examining individual patient profiles that graphically depict treatment and complication start and end dates, costs, as well as important PROMS, namely the summary score of the cancer-specific quality of life instrument QLQ-C30 [18] and derived utilities at BL, 3, 6, and 12 months. Utilities were derived from the QLQ-C30 summary score using the algorithm developed by Khan et al. [20], with corrections provided by Woodcock et al. [21]. A visual inspection of the profiles was conducted by the multi-professional team to verify, in a broad qualitative sense, the coherent integration of the individual components.

To validate the allocation of costs to specific treatments, we compared the number of treatments assigned to case numbers (based on our developed method) with the number of treatments originally recorded in the clinical dataset. We calculated summary statistics of costs by type of treatment (surgery, chemotherapy, radiotherapy, immunotherapy, targeted therapy), total costs per patient, and costs broken down by inpatient and outpatient services. We compared our cost estimates with results from the literature. Towards this end, we multiplied previously published results from other countries by the applicable purchasing power parity [22] and extrapolated them to 2023 values using the Swiss consumer price index [23].

We also calculated rank-based Spearman correlation coefficients to investigate a possible correlation between first-year costs per patient and utility change from baseline, by lung cancer histology and stage at diagnosis. For each patient, the last available utility measurement within the maximum 12-month patient FU was utilised. We did not replace missing baseline QLQ-C30 scores. Affected patients were excluded from the analysis of utilities.

To illustrate the opportunities for advanced analytics with the merged data, we fit partial correlations between costs and utility changes from baseline (S2 Fig in S1 Appendix), using the structural equation models (SEM) framework allowing simultaneous control for other factors (e.g., [24]). Constrained by patient numbers and length of FU in our interim data cut, the SEM analysis was restricted to histology and staging variables with sufficiently large sample sizes. Example results are presented for demonstration purposes only, with their value expected to increase as larger sample sizes and longer FU data become available in the final analysis.

All analyses were performed with the statistical software R (version 4.3.2).

## Results

### Lung cancer patient population

This cohort study enrolled 293 newly diagnosed lung cancer patients (S1 Fig in S1 Appendix). Among these, 62 were concurrently participating in clinical trials, 22 were from the Swiss cantons of Jura or Zurich, and 1 patient met both exclusion criteria. The derived FAS therefore consisted of 208 (71%) patients (S1 Fig, S7 Section in S1 Appendix). At the time of the analysis (November 24, 2023), 73 (35%) of the 208 FAS patients were dead (n = 36) or deactivated (n = 37). Deactivation reasons were personal concerns (n = 24), unresponsive FU (n = 9), health situation (n = 2), and other (n = 2). Among the 172 patients who had not died, mean FU duration was 10 months (n = 108, 63%, had a full 12-month FU after first diagnosis; n = 137, 80%, had a FU of 8-months or longer).

Most of the lung cancer patients in the FAS population had been diagnosed with adenocarcinoma (130/208, 63%) (S11 Table in S1 Appendix). The largest proportion of all patients (n = 89, 43%) was diagnosed with stage I, followed by 60 patients (29%) with stage IV disease. More than half of the patients were men (127/208, 61%) (S12 Table in S1 Appendix). The median age was 71 years, and the comorbidity distribution was as follows: 50/182 (28%) none, 4/182 (2%) low, 73/182 (40%) moderate, and 55/182 (30%) severe. Also, 39/179 (22%) of the patients had never smoked, 72/179 (40%) were ex-smokers, and 68/179 (38%) current smokers. Comorbidity information was missing for 26 (13%) of the 208 patients and smoking history for 29 (14%). S3 Fig in S1 Appendix outlines the first-year treatment sequence patients had received. There was one patient without any treatment before deactivation or death.

### Individual patient profiles and validation

**Fig 4:**
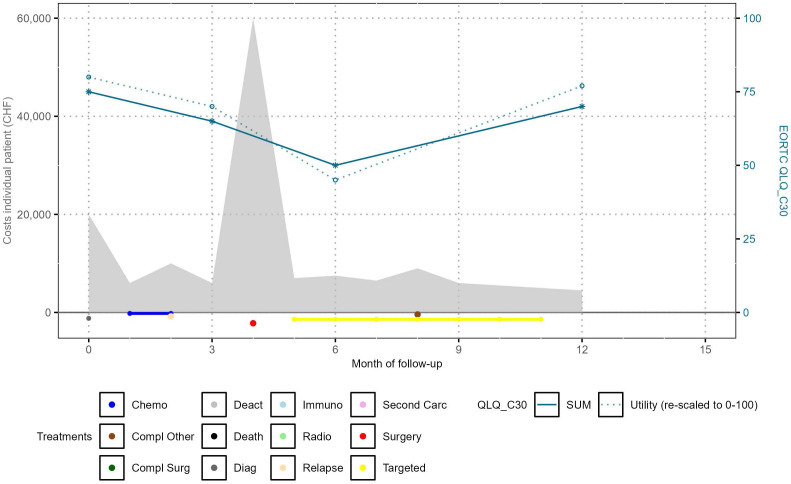
Fictitious profile of an example patient. Abbreviations: Carc carcinoma; chemo chemotherapy; CHF Swiss francs; compl complication; deact deactivation; diag diagnosis; QLQ quality of life questionnaire; SUM summary score of QLQ-C30. Comment: Costs were uniformly distributed within a case number over the period between the case number opening date and the case number closing date (measured in days) and presented monthly after the start of patient follow-up.

We found the concordance between treatments, complications, relapses, secondary carcinoma start and end dates, and cost peaks in individual patient profiles to be satisfactory. For data protection reasons, we can only show a fictitious patient profile (Fig 4). The graph shows the cost distribution as grey areas (related y-axis on the left), the EORTC-QLQ-C30 summary score and derived utility measurement [20] by blue lines (related y-axis on the right), and the treatment and event information from the clinical dataset in colour underneath the cost areas (directly above the x-axis label).

With the developed procedure, we were able to allocate each surgery (N = 130) to one single case number. For all radiotherapies (N = 97), we were able to allocate at least one case number per patient to radiotherapy (79 patients with at least one radiotherapy; 100% allocation). Radiotherapy was never co-assigned to the same case number as an inpatient surgery.

Based on the clinical dataset, we know that 87 patients had received chemotherapy. For 84 of these patients, we were able to assign chemotherapy to specific case numbers. However, for the remaining 3 patients (3%), no chemotherapeutic drugs could be identified in either the service or the cost dataset, preventing us from allocating chemotherapy-related costs for these patients. For immunotherapy and targeted therapy, allocation was not possible for 6 out of 61 patients (10%) and 6 out of 11 patients (55%), respectively. For a total of 13 out of 208 patients (6%), not all drugs used in systemic treatments could hence be identified. This may reflect drug names missing from our search strings, inconsistencies between datasets, or external purchases of oral therapies. Notably, targeted therapies may have been prescribed with the expectation of being purchased at an external pharmacy. Such external drug costs were, however, beyond our hospital perspective.

### Proof-of-concept results: first-year example results of merged data

#### Bringing costs into clinical context.

**Fig 5:**
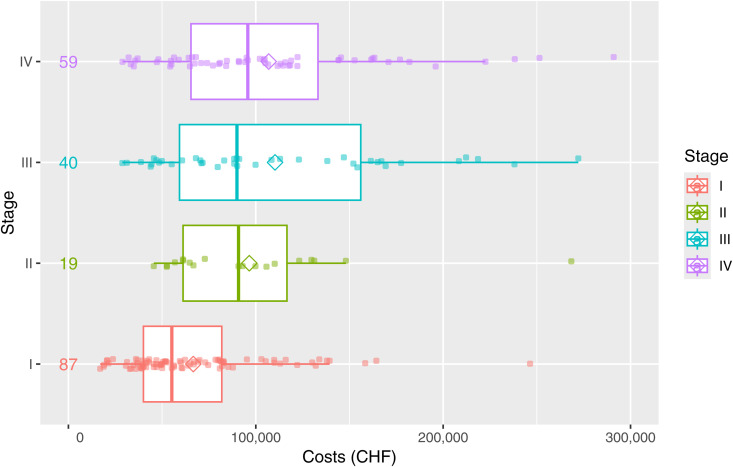
Total costs per patient and stage (maximum 1 year patient follow-up, FAS population). Abbreviations: CHF Swiss francs; FAS full analysis set. Comment: Extreme outliers > CHF 300,000 not shown.

Median total first-year costs per lung-cancer patient (regardless of disease stage at diagnosis and type of treatment) amounted to CHF 77,834 (mean CHF 93,621; standard deviation CHF 64,548). Minimum and maximum values were CHF 16,944 and CHF 473,160. We attributed about ¾ of the overall hospital costs to inpatient care and ¼ to outpatient care (S13 Table in S1 Appendix). The costs included treatment of comorbidities at USB during the observation period. Summary statistics of first-year lung cancer treatment costs per stage at diagnosis are shown in Fig 5. First-year costs for stage I tended to be lower than for later stages.

We were then able to assign costs to specific underlying therapies. For example, immunotherapy incurred the highest median costs per patient, of CHF 45,394, during the first year (mean CHF 49,518), followed by surgery costs of CHF 41,665 (mean CHF 48,940), and targeted treatment costs of CHF 31,522 (mean CHF 31,224) (Table 1). Patients who died in hospital had costs of CHF 31,437 for their last case number (mean CHF 38,518). First-year radiotherapy incurred median costs of CHF 16,684 (mean CHF 24,380), and diagnosis before the first treatment of CHF 11,901 (mean CHF 16,674). For the remaining “other” category, we derived median costs of CHF 7,846 per patient (mean CHF 18,186).

**Table 1:** Summary statistics of per patient hospital costs by cost type (maximum of 1-year patient follow-up, FAS).

Hospital costs	N^a^	Median (25%, 75% quantiles)	Mean (SD)	Min, Max
Diagnosis	173	11,901 (2,681; 22,479)	16,674 (18,752)	[0; 120,509]
Surgery	122	41,665 (33,552; 55,537)	48,940 (33,093)	[4,964; 332,792]
Radiotherapy	79	16,684 (9,075; 29,734)	24,380 (26,906)	[755; 157,454]
Systemic therapy	84	39,427 (13,183; 71,527)	47,858 (38,789)	[1,944; 196,241]
* Chemo*	*84*	*12,769 (7,720; 28,198)*	*19,765 (17,281)*	*[1,617; 86,829]*
* Immune*	*59*	*45,392 (23,759; 60,846)*	*49,518 (36,372)*	*[5,292; 196,241]*
* Targeted*	*5*	*31,522 (31,115; 32,349)*	*31,224 (10,280)*	*[16,067; 45,066]*
Other	192	7,846 (3,349; 20,422)	18,186 (33,033)	[249; 350,411]
Death in hospital	12	31,437 (19,951; 34,447)	38,518 (39,278)	[6,694; 154,042]
Total	208	77,834	93,621 (64,548)	[16,944; 473,160]

Abbreviations: FAS, full analysis set; N, sample size; Max, maximum; Min, minimum; SD, standard deviation.

^a^Costs of the same treatment type (e.g., chemotherapy or any other systemic therapy) were first summarised per patient. N stands for the number of patients with this treatment or service type.

#### Feasibility of correlation between first year costs and changes in utility.

**Fig 6:**
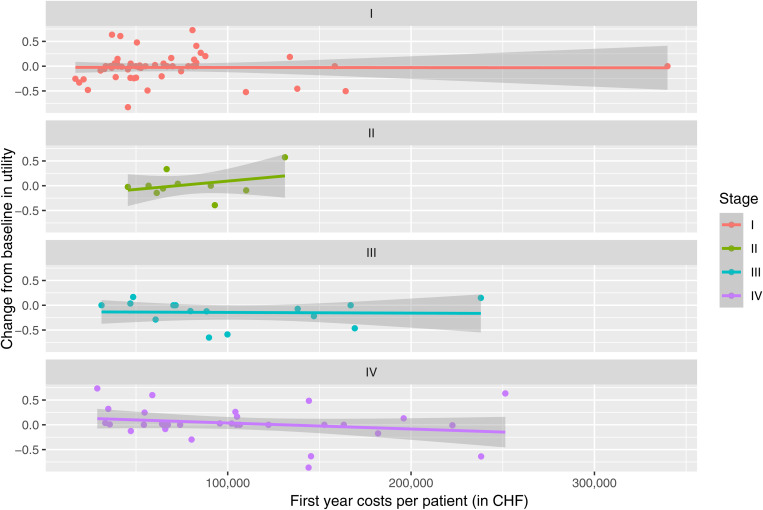
Association between first-year costs and changes in utility from baseline per patient by stage for adenocarcinoma patients. Abbreviations: CHF, Swiss francs.

Across different disease stages for the subpopulations of adenocarcinoma and squamous cell carcinoma patients, the example correlation results between total first year costs and utility change from baseline varied markedly in terms of the strength of the correlation and their direction (Fig 6, S4 Fig S4, S14 Table in S1 Appendix).

Among adenocarcinoma patients in stage IV, a negative partial correlation of −0.307 was observed between first year costs and utility change from baseline. This finding, derived from a small sample size (n = 32), is intended solely to illustrate the potential analytical approach. If this observation was replicated in a larger sample, it could suggest that after accounting for baseline utility and the type of first treatment, higher total first-year costs might be associated with a decline in utility from baseline. In contrast, the partial correlation for patients with squamous cell carcinoma in stage IV was positive (with a result of 0.821), meaning that higher total first-year costs could be associated with an increase in utility from baseline, again adjusting for baseline utility and the type of first treatment. The latter outcome was however based on data of 7 patients only, yielding to the merely exploratory and exemplary nature of the current correlation results. For substantially larger population sizes, similar analyses might be applied to optimise first and further-line therapies for patients with similar baseline utilities.

## Discussion

The VBHC concept is becoming increasingly important as healthcare systems strive to optimise patient outcomes while controlling costs. Many hospitals still lack standardised processes and systems to measure and evaluate patient outcomes and to bring them into context with costs across various treatment modalities and time. Even where complete patient data are available, they are currently stored in disparate systems not intended for seamless data integration, making complex data analytics for the merging process necessary. In Switzerland, a crucial point is that the highly standardised hospital financial reporting and billing system does not allow one to infer what each therapy costs. The focus is on costs per ward, centre, or partner, but not on costs per “overarching” treatment like, e.g., chemotherapy. Our study addresses these real-world limitations in the implementation of VBHC by demonstrating the need for further development in data collection, processing, and storage at the service provider level (meso- and micro-level). It also demonstrates the feasibility of using routinely collected patient-reported outcomes and combining the so defined patient benefit with costing data, which can serve as a foundation for the discussion on a national level with healthcare leaders and decision makers (macro-level).

We have successfully developed a scalable method for linking CROM and PROM data (collected according to ICHOM standards) with service and cost data from the Swiss hospital administrative datasets (REKOLE^®^). The method is based on the concept of case numbers (used for financial reporting and billing purposes), treatment and service dates, and targeted search queries. We propose an approximation method to break down the total hospital costs for lung cancer patients, separating expenses for the different types of treatment (for example surgery, chemotherapy) as well as for comorbidity care. We successfully demonstrated that the method can provide individual patient profiles simultaneously visualising treatments, costs and PROMs over time. Our method demonstrated to be reliable by assigning surgery and radiotherapy treatments to all patients who received these treatments. Most systemic therapies (94%) could be assigned to the patients undergoing those therapies. One limitation was reliable assignment of targeted therapy (45%), which may be attributable to the chosen hospital perspective. This perspective does not account for oral medications sourced from an external pharmacy and taken at home.

Our proof-of-concept allowed us to derive, as an example, first-year lung cancer costs, overall and by treatment type (total median costs per patient CHF 77,834; mean CHF 93,621). We assume the category “other” with median costs of CHF 7,846 per patient (mean CHF 18,186) to represent a mix of costs for lung cancer unrelated comorbidity treatment and lung-cancer-relevant medical FU services that could not be allocated (S15 Table in S1 Appendix). The remaining CHF 69,988 (out of the overall CHF 77,834) may thus be interpreted as the median first-year costs per patient that are incurred as a minimum for the treatment of lung cancer without treatment of comorbidities.

Recent literature on lung cancer treatment costs is sparse and available cost results strongly fluctuate [25]. The latest available publications are based on data that is at least 5 years old and tend to show highly variable cost results, usually lower than ours [26–35]. Differences in costs across publications are of course to be expected given differences in study design, patient selection, costs and costing methods, medical practice differences, price levels, and price years. For example, a recent and detailed Italian whole-disease model [26] estimated first-year mean direct medical costs of CHF 39,518 per non-small cell lung cancer patient, derived from 2019 data using literature-based probabilities and a payer perspective [22,23]. In Japan, median first-year lung cancer costs derived from 2017 data were estimated at CHF 30,059 [22,33,36]. In a Swiss publication based on 2011 data [34], mean direct medical first-year costs for lung cancer treatment were estimated between CHF 63,727 and CHF 76,917 per patient after inflation to 2023 values. This is consistent with our results.

We cannot draw conclusions about correlations between first-year hospital costs of lung cancer patients and utility changes from baseline, due to the small sample sizes in the subgroups formed by histology and stage at diagnosis, and the short patient FU. Structural equation models may be applied in the future when case numbers are sufficiently large, allowing for more robust analyses of the relationships between patient treatments, costs and PROMs. However, example correlations were performed to demonstrate the potential of a larger roll-out of the presented approach.

### Strengths

The main strength of our approach is that it reflects the real-world setting and that the required data are consistently available across Swiss hospitals, as they are collected in line with the Swiss routine hospital financial reporting system and standardised nomenclature of ICHOM (or similar standardised PROM and CROM datasets). Furthermore, the method is replicable by other Swiss hospitals or by other European hospitals (e.g., in Germany) with PROM measurements as a prerequisite and similar data structures. In the United States (U.S.), the Centres for Medicare & Medicaid Services use a cost-measurement system which is inferior and less granular than the systems used for internal cost management in many hospitals [37]. Ederhof et al. recommend adopting the Swiss REKOLE system as a standardized and synchronised cost-measurement system also in the U.S., for both DRG payment setting and internally for hospitals. This could enable the transferability of our approach to the U.S. context upon its implementation.

A second important strength of our study is the low percentage of data inconsistencies in the available USB real-world service and cost dataset (as detailed in S5 Table in S1 Appendix) that allowed us to successfully conduct our pilot analysis. However, a higher level of inconsistencies could translate into significant limitations, potentially jeopardising the planned analyses.

Next to delivering the necessary data foundation for the further development of VBHC, individual patient profiles can be extracted as a standard graphical tool for use in routine clinical care of lung cancer patients, to monitor patient treatment and benefit over time while controlling costs.

### Limitations

Our assessment faced several challenges. Most obviously, the real-world data sources used were developed for other purposes, so that combining them was complex. However, we managed to address most uncertainties by reasonable assumptions that allowed an approximative cost-to-treatment assignment. Simplifying assumptions were required to assign costs to several treatments covered by the same case number. We believe that an even closer collaboration and involvement of experts from finance departments would allow us to leverage further information to refine the assignment of hospital costs to specific clinical data.

Another limitation was the frequent trial participation of patients at a university hospital as well as referral agreements between hospitals. Both situations generate incomplete data and lead to unknown influences on the results of the affected patient population, potentially introducing bias. The extent to which the inclusion of such patients may influence overall analysis results can be evaluated through sensitivity and scenario analyses in larger real-world datasets, and by comparing how trial populations differ from the general patient populations.

We believe that our estimated first-year costs per patient would likely have been higher if all patients had received a full 1-year FU, and if the scope of the analysis had been broadened to a healthcare system perspective (including lung cancer drug costs incurred outside the hospital setting). However, due to the small number of patients with a high probability of hospital-external targeted treatment (n = 6), we assume the external targeted drug costs only had a small influence on our overall cost results per patient. Overall, the total number of 11 patients with targeted therapy in our pilot analysis was low, limiting the conclusions that can currently be drawn about this type of treatment.

For the application of the new approach in prospective frameworks, we currently identify one main limitation: the delayed availability of costing data, often by several months. Nevertheless, this may evolve in the future with improvements in data timeliness. Should such advancements occur, our merging strategy would also be well-suited to support timely and efficient prospective analyses.

### Recommendations and next steps

The quality of the results that can be achieved by merging PROM and CROM, service and financial datasets are largely dependent on the accuracy of all three datasets. In particular, the quality of the derived cost estimates is subject to the quality of recorded clinical data, particularly with respect to treatment start and end dates. In our case, PROM and CROM data were entered manually into the Heartbeat^®^ system by two study nurses. To minimise the risk of typing errors inherent in manual data entry, implementing pre-programmed, automated data validation checks can enhance data quality while saving time. Additionally, our methodological approach could be simplified by directly extracting case numbers alongside clinical data from medical records. Adopting structured and standardised data collection systems in hospitals would further enhance data reliability and adaptability, enabling its efficient use for a wide range of analytical purposes. Moreover, early input and collaboration with data management, statistics, and finance teams is recommended to set up such systems but also the analyses they support.

While costs could be linked to individual treatments (e.g., surgery, immunotherapy), a subsequent challenge would be breaking down and evaluating costs by entire treatment strategies across the full cycle of lung cancer care.

Further steps could include replicating the new approach in other Swiss hospitals, incorporating externally sourced immune and targeted drug costs, and performing detailed and longer-term analyses of population-based interrelationships between costs and benefits which drive VBHC. The latter, however, requires larger datasets than those currently available to us. Above all, this holds for the partial correlation analyses which should be repeated and extended after a future data transfer. Next to QLQ-C30 questionnaire data, other PROMs or also clinical endpoints could be added into the VBHC equation to further enrich the analytical potential and to optimise several patient benefit criteria simultaneously while controlling costs.

Shared decision-making relies on timely and transparent access to patient-reported outcomes and clinical data, enabling patients and providers to jointly select the most appropriate care options. Similarly, care pathway redesign benefits from integrated data by identifying variation in outcomes and resource use, thus informing evidence-based improvements. Data integration also lays the groundwork for innovative reimbursement models, such as bundled payments or outcomes-based contracting, by ensuring that performance metrics are measurable and comparable. This, in turn, may help prevent “cherry-picking of services based solely on profitability criteria”, especially related to DRG inpatient payments [38]. DRG systems are implemented in numerous European countries, as well as in the United States, Canada, and several Latin American nations. By aligning data systems with these broader reforms, healthcare organizations can better realise the full potential of VBHC.

The foundations laid by this work are not yet sufficient to initiate a reimbursement reform. For this to happen, data would have to be collected from multiple hospitals and show an improvement over time, attributable to VBHC, both in terms of outcomes and costs. This can only be initiated in a subsequent step. Our approaches may be used, in a first step, for shared decision-making. Thanks to the method developed here, it is possible to add and evaluate the PROMs and cost data at any time in order to make improvements to the patient pathway.

## Conclusion

Our innovative methodological merging approach enables combined analyses of PROMs and CROMs, and financial information and represents a significant stride towards a VBHC model. By visualising individual patient pathways, as exemplified in Fig 4, and generating the potential for comprehensive population-based analyses using merged datasets, it may serve as a cornerstone for patient-centric care and quality improvement initiatives at the level of institutions but also at a system-level.

In comparison with the literature, our methodological approach delivers consistent results, reinforcing our confidence in its application and potential for future development. The generated datasets are curated and will allow further analyses with traditional and more sophisticated statistical models when data from more patients and with a longer observation period become available.

The Swiss healthcare system has been confronted with continuously rising expenditures and a growing shortage of specialists over the past decade. The system could therefore benefit substantially from the type of efficiency gains our methodology may help to identify. The resulting information may support the efforts of decision-makers at both the hospital and political levels, as well as assist payers’ decisions.

## Supporting information

S1 Appendix S1–S4 Figs, S1–S7 Sections, S1–S15 Tables. (PDF)
