# Supplementary material for: Contributing to a value-based health care framework for lung cancer patients in Switzerland – A methodological approach to merge routinely collected hospital data
Source: PLoS One. 2025 Jul 10;20(7):e0327814. doi: 10.1371/journal.pone.0327814 (PMC12244553; doi:10.1371/journal.pone.0327814)
Supplement: S1 Appendix — (PDF) [file pone.0327814.s001.pdf]

Supplementary Appendix

Contributing to a value-based health care framework for lung cancer patients in Switzerland – merging routine level hospital datasets

Michaela Carla Barbier[1,2,*], Katya Galactionova [1][3], Mark Lambiris[1,2], Leonel Oliveira[4], Florian Rüter[4], Dominik Glinz[5], Jessica Thürmer[5], Flurina Pletscher[5], Benjamin Kasenda[6], Tobias Finazzi[7][8], David König[6], Didier Lardinois[9], Larissa Conrad[10], Leonie Mutz[4], Matthias Schwenkglenks[1,2]

[1] Institute of Pharmaceutical Medicine (ECPM), University of Basel, Basel, Switzerland

[2] Health Economics Facility, Department of Public Health, University of Basel, Basel, Switzerland

[3] F. Hoffmann-La Roche AG, Basel, Switzerland

[4] Quality Management & Value-based Health Care, University Hospital Basel, Basel, Switzerland

[5] Roche Pharma (Schweiz) AG, Basel, Switzerland

[6] Medical Oncology, University and University Hospital Basel, Basel, Switzerland

[7] Clinic of Radiotherapy and Radiation Oncology, University Hospital Basel, Basel, Switzerland

[8] Department of Radiation Oncology, Cantonal Hospital Baden, Baden, Switzerland

[9] Department of Thoracic Surgery, University Hospital Basel, Basel, Switzerland

[10] Medical controlling, Finance Department, University Hospital Basel, Basel, Switzerland

Content

A) Appendix figures

B) Appendix sections

C) Appendix tables

A) Appendix figures

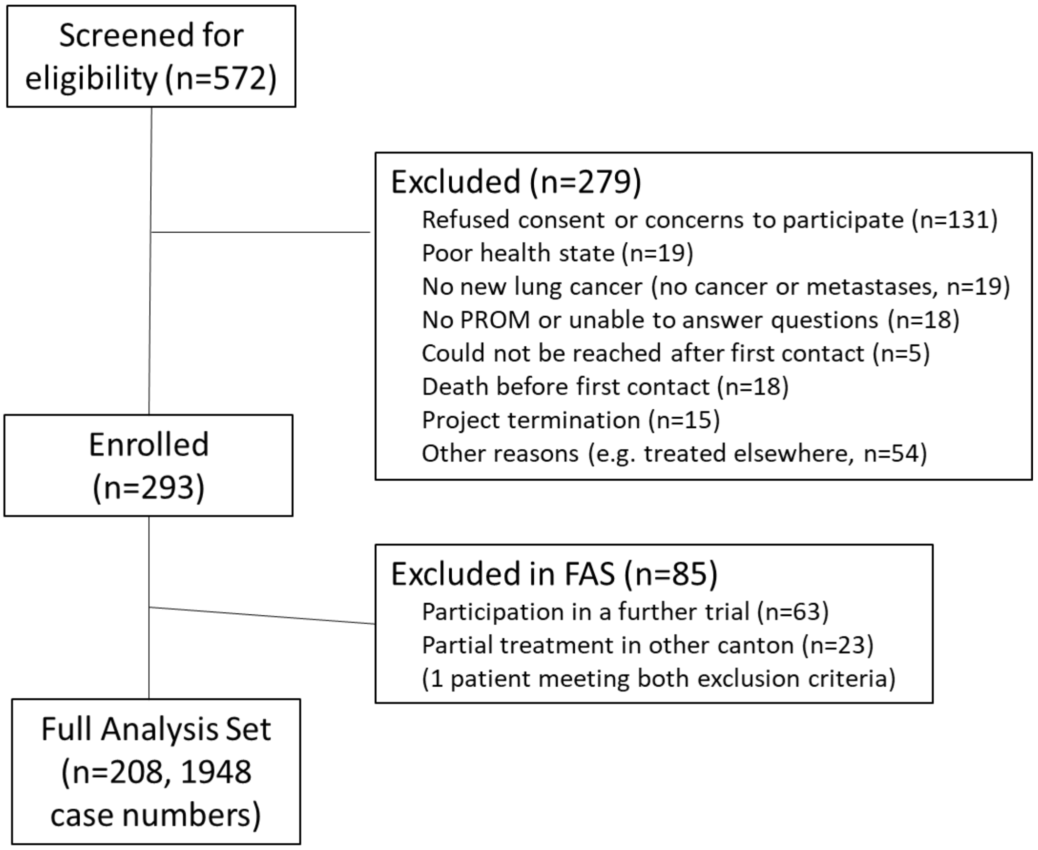

S1 Fig. Patient flow chart.

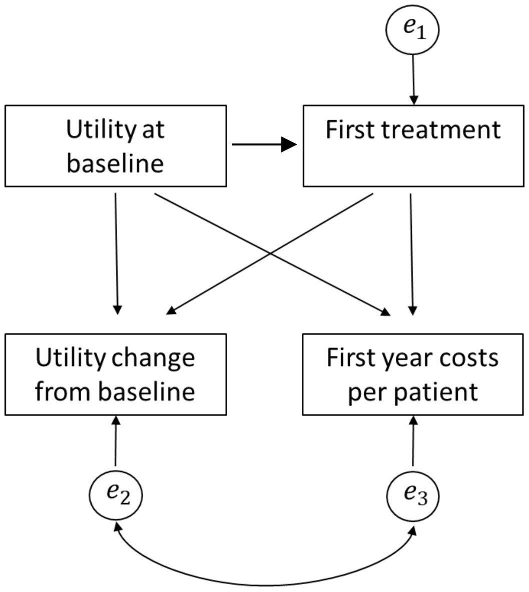

S2 Fig. Partial correlation model to investigate a noncausal association between utility change from baseline and first year costs per patient.

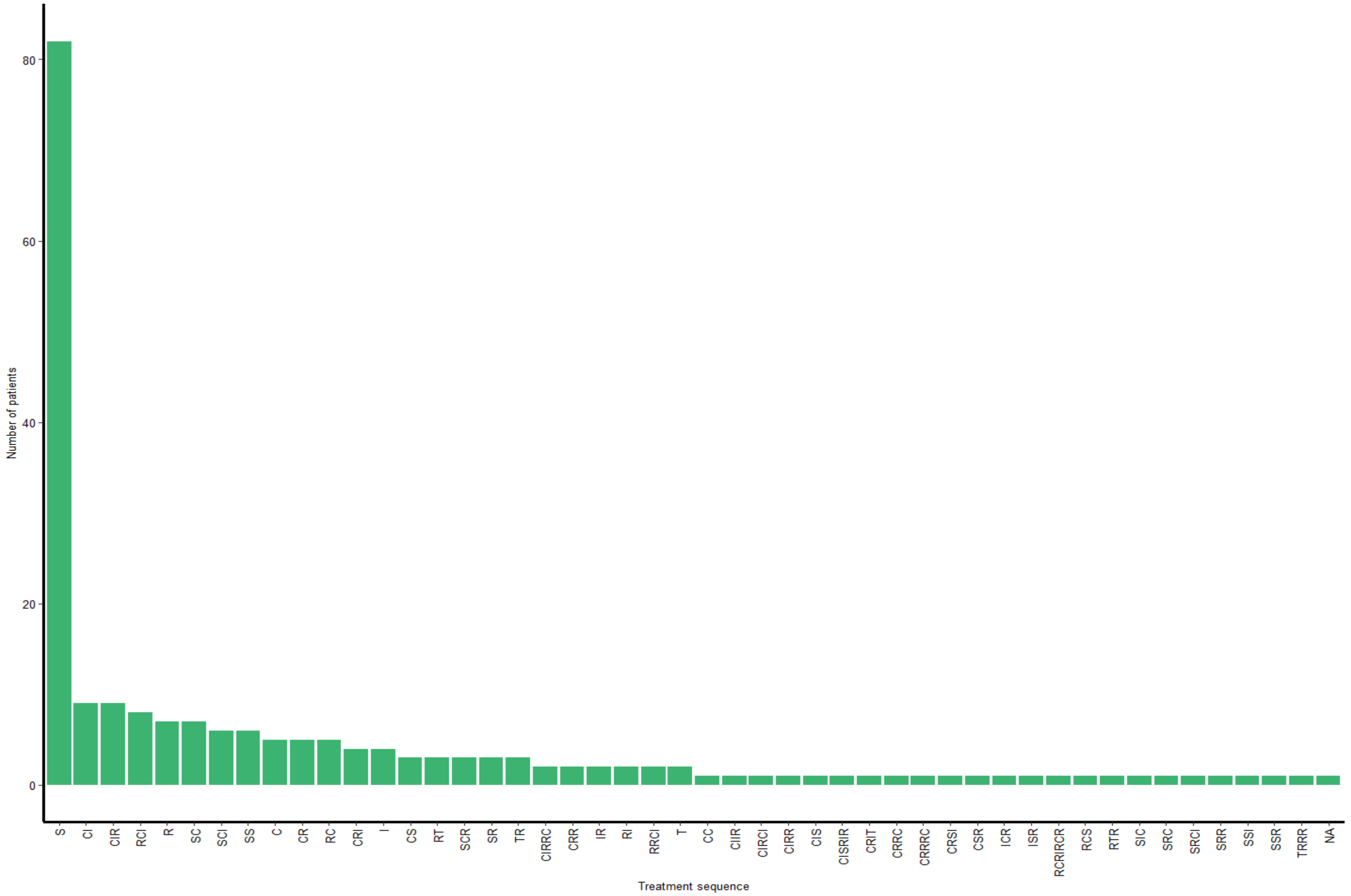

Abbreviations: C Chemotherapy; I Immunotherapy; NA not applicable (1 patient without any treatment); R Radiotherapy; S Surgery; T Targeted Therapy

S3 Fig. Distribution of patients by first year treatment sequence (Full analysis set).

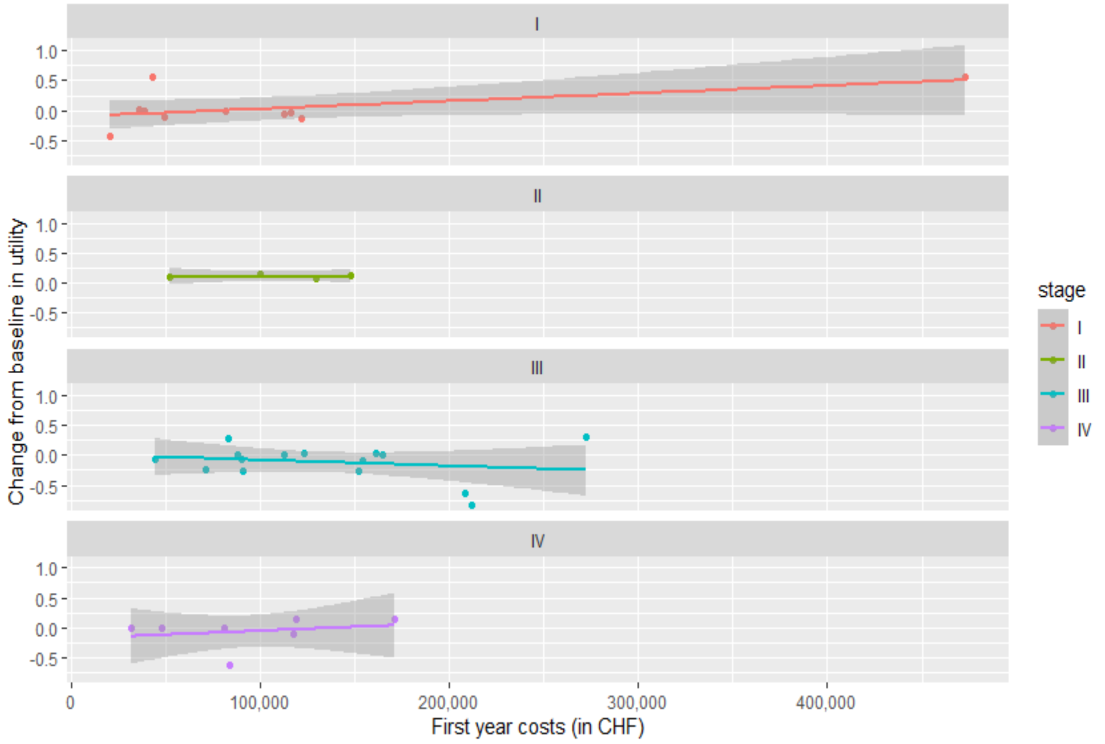

S4 Fig. Association between first year costs and changes in utility from baseline per patient by stage for squamous cell carcinoma patients.

B) Appendix sections

S1 Section. Patient population

The analyses presented in this paper are based on a maximum patient followed-up of one year, with claims allowed from 11 days before the diagnosis date and up to the date of either death, deactivation, 1-year after diagnosis, or the interim cut date (24. November 2024), whichever came first. The extension to 11 days before diagnosis was chosen to enable the inclusion of services and costs directly related to lung cancer diagnosis. A typical example is a procedure to obtain tissue material that only subsequently leads to a cancer diagnosis.

S2 Section. Comorbidities

Comorbidities were defined in the HEARTBEAT® database as follows:

Value	Description
0	I have no other illnesses
1	Heart disease (for example angina, heart attack, heart failure)
2	High blood pressure
3	Pain in the legs when walking due to poor circulation
4	Lung disease (e.g. asthma, chronic bronchitis, emphysema)
5	Diabetes mellitus
6	Kidney disease
7	Liver disease
8	Consequences of a stroke
9	Disease of the nervous system (e.g. Parkinson's disease, multiple sclerosis)
10	Other cancer (in the last 5 years)
11	Depression
12	Arthrosis/arthritis

We transformed the comorbidity variables into an age-adjusted comorbidity score with the levels high, medium, low, and none, using the algorithm provided in Charlson et al. [1] and Colinet et al. [2].

S3 Section. Additional variables in the service dataset

A very large number of service claims of different service types and tariff types at different time points are usually recorded with the same case number and can cover the entire range of possible services (for example physician consultations, laboratory and imaging services, disposable materials, drugs, implants,). Each individual service claim within a case number is accompanied by a textual description of the service (service text), and exact dates (admission date, service date, discharge date).

S4 Section. Data particularities, data transfer, and data validation

In general, differences in the structure of the datasets present a challenge for linking.

Clinical dataset versus cost dataset:

The clinical dataset records for each patient information on treatment type (e.g. surgery, chemotherapy), treatment date (start and end dates), and complication type and date. In contrast, several cost centres, hierarchies, organisational units, and a month and year in which the cost is billed are assigned to costs

within a case (without a patient identification number) in the cost dataset. There is no link between treatments and cost centres nor is there a direct link between the date of treatment and the month of billing. For inpatient services, international classification of diseases (ICD) and Swiss diagnosis related group (DRG) codes in the cost dataset may only partly be of help for the assignment of costs to surgeries. Otherwise, ICD and DRG codes also do not allow the linking of an obtained treatment (like e.g. a chemotherapy) to a related battery of cost claims.

Clinical dataset versus services dataset:

There is also no direct mapping possible between treatments and services. Any treatment for which there is only a start and an end date available in Heartbeat® may involve a large battery of services that could be delivered across various hospital departments and at different individual dates, obscuring the alignment between the clinical and the service dataset. Consider, for instance, any surgical treatment that may involve, besides the surgeon and the surgical ward, anaesthesiologists, nurses, laboratory, etc..

Services dataset versus cost dataset:

Although they often have similar entries, the variable service and tariff type in the service dataset are not directly compatible with any of the cost type, cost centres and hierarchy variables of the cost dataset. A direct linking of the data in the services and cost dataset via these variables is therefore not possible. The textual description of the services in both the service and the cost dataset does also not allow direct cross-linking with an obtained treatment due to the missing service date in the cost dataset.

Data export and data validation

The transfer of all datasets followed all cantonal and national data protection regulations performed via a password-protected "Secure Transfer" from the University Hospital Basel (USB) Quality Management & Value-based Health Care (QM & VBHC) department to the European Center of Pharmaceutical Medicine (ECPM) for merge and analysis. The USB QM & VBHC department was responsible for data quality of the clinical dataset, and the USB finance department for the services and cost datasets.

S5 Section. Merging strategy step 1

Starting with the patient identification number and dates in both the clinical and the service dataset (clinical dataset: treatments, complications, patient-reported outcome measure (PROMS) dates; service dataset: service dates), we extracted the case numbers from the service dataset corresponding to services whose service dates fell into our follow-up period. Missing service dates were replaced by the first service date within the relevant case number, and if this date was also missing by its last service date. We excluded service claims with no dates at all from our analysis (~0.1%, **supplementary Table S5**). In the next step, we reduced the cost dataset to the list of retained case numbers. We summed the costs of all services within each case number. The rationale behind this approach was that each case number is generally related to an overarching treatment (a single treatment such as a surgery or a treatment combination such as radio-chemotherapy).

S6 Section. Assignment of treatments to case numbers in the grouped dataset

The search procedure necessitates its application in the following order outlined below. We prioritised surgery and radiotherapy in our procedure as both treatments could clearly be assigned to case numbers (surgery as an inpatient service was easiest).

1) Surgeries

To identify case numbers related to surgeries in the grouped dataset, we first assumed that lung cancer surgeries are performed in an inpatient setting only. Start and end dates of candidate case numbers also needed to include a patient's surgery date [CASE_starting_date <= surgery_date <= CASE_end_date]. Hence, we only selected inpatient cases for patients with confirmed surgery and recorded surgery dates (based on the clinical dataset) within the opening and closing of a case. In addition, at least one receiving, executing, or discharging nursing or specialist units should have been "Thoraxchirurgie", or the executing nursing unit should have been "operation room" ["2003 – operationssäle"]. Besides, we searched for relevant strings in the service text [gve02c|gve05a|gve05b|kte02c|kte05a|kte05b|zve05b] or obliged the service_type to contain one of the three options: "opsl – operationsleistung", "drg – ltyp", or "implantate". With this search strategy, we were able to assign all surgeries to a single case number.

To relate also subsequent ambulant service claims to an obtained inpatient surgery, we checked among patients with a confirmed surgery if both executing and discharging nursing units were "thoraxchirurgie" in the service dataset or if the executing nursing unit was "thoraxchirurgie" in the cost dataset. We also assigned such cases to surgery.

2) Radiotherapies

We subset the cost and the service dataset to patients who had received at least one radiotherapy (based on information from the clinical dataset).

In the cost dataset, we further retained all case numbers that had the cost_types *"2800 - nuklearmedizin und radioonkologie al"* or *"2810 - nuklearmedizin und radioonkologie tl",* assuming that only radiotherapy services would be billed with this cost type. We additionally totalled up costs of these claims only (within a case number) in the event that a split between different treatment types (e.g. radio- and chemotherapy) was appropriate within a case number.

In the service dataset, we looked for certain keywords in the variable "service text" which were: "hochvoltstrahlentherapie | hochvolt | strahlentherapie | strahl | beschleunigertherapie | bestrahl", but NOT "ohne strahlentherapie| röntgen | dickdarm | lobektomie". Related English terms are "high voltage radiation therapy", "acceleration therapy", but not one of the following expressions "without radiotherapy"," without radiation therapy", "X-ray", "colon", "lobectomy").

The common list of identified case numbers from both datasets represented our final list of case numbers allocated to radiotherapy.

3) Systemic treatments

To link systemic treatments to case numbers, we searched for drug names in the descriptive variable "service text" in both the services and cost dataset. The list of drug names is given in **Table S8**. Systemic treatment start and end dates were not used as a further restriction next to drug names, as we assumed that identified chemotherapy, immunotherapy, and targeted therapy from our medication list were in any case of relevance to our FAS population even if not indicated in the clinical dataset. The hits in the cost and service datasets slightly differed. The service dataset provided 7 additional treatment assignments (although without costs). We attributed the differences in the common variable "service text" in the two real-world datasets to data inconsistencies.

It happened several times that multiple treatments were identified and assigned to the same case number (e.g. chemotherapy and immunotherapy or chemotherapy and radiotherapy).

Although leading to the same result, the runtime of the computer program could considerably be reduced by further subsetting in the above-mentioned search query cost_type to "4000 – Arzneimittel" in the cost dataset, and service_type to "medi - medikamente ohne tagesdosis" or (service_type to "drg - ltyp 1 drg" & tarif_type to "011 – zusatzentgelt") in the service dataset.

4) Relating case numbers to "death in hospital" if not attributed differently

Death date and death location (1=hospital, 2=home residency, 3=retirement or nursing home, 4=hospice, 888=other) was stored in the clinical dataset. In the event that a patient had died in hospital (death location = 1), the death date was within the case opening and closing dates, and the respective case number had not been assigned yet, we allocated "costs of death in hospital" to this case number. Out of overall 17 deaths in the FAS population, we assigned all 12 still open case numbers to death in hospital. We verified that the remaining 5 deaths had happened within a case already allocated by a single or combined treatment (twice chemo-radio, once chemo-immune, once radio-immune, once surgery).

5) Separate diagnosis before first treatment (if applicable)

Based on the diagnosis date and the first treatment date (HEARTBEAT® system), we assigned all outstanding cases to "diagnosis" that had occurred within the time interval of window (11) days before the diagnosis date and 1 day before the first treatment date. For the one patient without any treatment, we assigned all cases to diagnosis.

6) Remaining open case numbers

All other open cases were in the following attributed to the category "Other" and incorporated comorbidity treatment, follow-up services of lung cancer treatment without a billed drug, or possibly complications, relapses and secondary carcinomas related to lung cancer treatment but not already captured in other case numbers.

S7 Section. Analyses sets, data transfer and data validation

Full-analysis set:

In terms of programming, we identified patients from the canton Jura based on a hit in the search string "jura" in any of the receiving, executing or discharging unit variables in either the cost or the service dataset (n=16 patients). Furthermore, we excluded n=6 additional patients from the canton Jura (n=6) or Zürich (n=1) with information found in their electronic health records.

C) Appendix Tables

S1 Table. Simplified snippet of the original clinical dataset in its long format (ICHOM defined PROMs and CROMs stored in the HEARTBEAT® system)

Patient ID	TYPE	DOCUMENT ID	EVENT	ATTRIBUTE ID	VALUE of ATTRIBUTE ID
...	*Anamnesis or any of the following factors: Patient QLQ-C30, QLQ-LC29, Baseline, Complications, Survival FU, Treatment*	*Number that stays the same for all variables measured at the same time point for an individual patient. Document_id varies for different measurement timepoints*	...	*Individual Case mix variables like e.g. diagnosis date, weight loss, smoking, comorbidities, FEV1, TNM stage,, ...*	...
1234567	Patient	a1811c7c-6d00-4abc-af2a-a1a52139baaa		Age	60
1234567	Patient	a1811c7c-6d00-4abc-af2a-a1a52139baaa		Sex	M
1234567	QLQ_C30		Baseline	QLQ_C30_SUM	75
1234567	QLQ_C30		3M FU	QLQ_C30_SUM	50
1234567	QLQ_C30		6M FU	QLQ_C30_SUM	25
1234567	Treatment		Treatment	Surgery	Yes
1234567	Complication		Complication	Complication Surgery	1
1234567	Survival		Survival FU	Relapse	1
1234567	Survival		Survival FU	Relapse date	01/03/2013
1234567	Survival		Survival FU	Death date	01/01/2015
...	...	...	...	...	...

Abbreviations: CROM clinician reported outcome measure; FEV1 forced expiratory volume; FU follow-up; International Consortium for Health Outcomes Measurement ICHOM; ID identifier; M month; PROM patient-reported outcome measure; QLQ quality of life questionnaire

Please note that the clinical dataset did not provide information about which treatment triggered which complication.

CROM variables included:

1) Baseline sociodemographic and medical history (e.g. year of birth, sex, highest education level, smoking history, weight-loss, comorbidities)
2) Baseline clinical assessment (among others: forced expiratory volume in 1 second (FEV1), forced vital capacity (FVC), single-breath diffusing capacity for carbon monoxide (DLCOSB), Eastern Cooperative Oncology Group (ECOG) performance status, TNM staging, driver mutations, histology)
3) Date of lung cancer diagnosis
4) Treatment information and related dates (surgery, radiotherapy, chemotherapy, immunotherapy, targeted therapy)
5) Complication types and dates (surgery related, other treatment related)
6) Survival and relapse follow-up. Tumour board-recommended combination treatment was possible (e.g. chemo-radiotherapy, chemo-immunotherapy)

S2 Table. Snippet of derived clinical dataset with a focus on treatment information that we want to assign to different case numbers

EHR_ID (=Patient identification number)	Treatment	Start date	End date
1111115	Diagnosis[1]	01/01/21	15/01/21
1111115	Complication cytopenia	06/03/21	06/03/21
1111115	Radiotherapy	23/05/21	10/06/21
1111115	Chemotherapy	xx/xx/xx	xx/xx/xx
1111115	Immunotherapy	xx/xx/xx	xx/xx/xx
1111115	Death		xx/xx/xx
1111118	Diagnosis	xx/xx/xx	xx/xx/xx
1111118	Surgery		xx/xx/xx
1111118	Deactivation		xx/xx/xx
.....			

[1] If applicable, the diagnosis end date was set to the day before first treatment start

Abbreviations: EHR electronic health record; ID identification number

S3 Table. Original services dataset

Original variable names in German	Variable names in English	Example entries in German *or further comments*	Used for merge and treatment assignment
Patient.Nr	Patient ID	2222001	Yes
Fall.Nr	Case number	88800011	Yes
Aufnahmedatum	Admission date	*Official case number opening date*	Yes
Entlassungsdatum	Discharging date	*Official case number closing date*	Yes
Leistungsdatum	Service date	*Date medical service was delivered/performed*	Yes
Leistungstyp	Service type	DRG- LTYP 1 DRG<br>LABO – Laborleistung<br>MEDI – Medikamente ohne Tagesdosis<br>MELE – Medizinische Leistung<br>OPSL – Operationsleistungen<br>RADI – Radiologieleistungen<br>Statistik - ziffer für co statistik<br>...	Yes
Tariftyp	Tarif type	001 – Tariftyp TARMED<br>010 – SwissDRG<br>011 – Zusatzentgelt<br>400 – Medikamenten-Katalog (Pharmacode, 7st)<br>402 – Medikamenten-Katalog (EAN-Code)<br>403 – Medikamenten-Katalog (Swissmedic-Nr.)<br>STATISTIK – Statistische Leistung	Yes
Fallart	Case type	Ambulant/day clinic, ambulant, inpatient,	Yes
Aufne. fachl. OE.Nr.Text	Receiving organisational specialist unit		Yes
Aufne. pfleg. OE.Nr.Text	Receiving organisational nursing unit		Yes
Erbr. pfleg. OE.Nr.Text	Executing organisational nursing unit	4660 - Chirurgie 6.1<br>*(Comment: variable changes within the same case number)*	Yes
Entl. fachl. OE.Nr.Text	Discharging organisational specialist unit		Yes
Entl. pfleg. OE.Nr.Text	Discharging organisational nursing unit		Yes
Leistung.Nr.Text	Service text	(Blanks)<br><br>00.0010 - Konsultation, erste 5 Min. (Grundkonsultation)<br><br>9023186 - DAFALGAN Tabl 500 mg 100 Stk<br><br>9024035 - NACL Fresenius Inj Lös 0.9 % 10ml PP Amp 20 Stk<br><br>9044374 - RAPIDOCAIN 50 mg/5ml o Kons 10 Amp 5 ml<br><br>9058933 - Biopsiezange RAD JAW 3 Pulmonary 1521	Yes
Leistungseinheit	Service unit	MG, ML, ST, ..., (Blanks)	No

Abbreviations: Aufne aufnehmende (receiving); DRG diagnosis related group; Entl entlassende (discharging); Erbr erbringende (executing); ID identification number; Nr Nummer (number); OE Organisationseinheit (organisational unit); MG milligram; ML milliliter; Stk Stück (piece)

S4 Table. Original cost dataset

Original variable names in German	Variable names in English	Example entries	Used for merge and treatment assignment
Fall.Nr	Case number	88800011	Yes
Fall.Zusammengeführt	Merged case number		No
REK.Hier_2	Rekole hierarchy 2	Individual costs ("Einzelkosten")	No
REK.Hier_4	Rekole hierarchy 4	380, 400, 401, 405, 480, 486	No
H_LEVEL_KOSTENSTELLE_ LEVEL_5_ID_TEXT	H_level_CostCenter (cost_level_5)	LABO – Labormedizin<br>NUKL – Nuklearmedizin<br>ONKO – Onkologie<br>RAON - Radioonkologie<br>THCH - Thoraxchirurgie	No
Partner.Kostenstelle.Nr.Text	Partner.CostCenter. Nr.Text (cost_partner)	303001.31 – Thoraxchir Ärzte sta<br>303002.31 – Thoraxchir Ärzte amb<br>304502.39 – Chirurgie 4.1<br>304503.39 – Chirurgie 5.2	No
REKOLE.Zeile.NrText	REKOLE.Row. NumberText (called cost type)	4000 - Arzneimittel	Yes
Fallart.Text	Case type	Ambulant/day clinic, ambulant, inpatient	Yes
Aufne. fachl. OE.Nr.Text	Receiving organisational specialist unit		Yes
Aufne. pfleg. OE.Nr.Text	Receiving nursing organisational unit		Yes
Erbr. pfleg. OE.Nr.Text	Executing organisational nursing unit	*Variable is always the same as the discharging organisational nursing unit*	No
Entl. fachl. OE.Nr.Text	Discharging organisational specialist unit		Yes
Entl. pfleg. OE.Nr.Text	Discharging organisational nursing unit		Yes
Leistung.Nr.Text	Service text	0009023186 - DAFALGAN Tabl 500 mg 100 Stk<br><br>0009024035 - NACL Fresenius Inj Lös 0.9 % 10ml PP Amp 20 Stk<br><br>0009044374 - RAPIDOCAIN 50 mg/5ml o Kons 10 Amp 5 ml<br><br>0009058933 - Biopsiezange RAD JAW 3 Pulmonary 1521<br><br>0009060579 - GEFÄSSPROTHESE 30CM 16MM GORE K<br><br>0009086268 - PRIVIGEN Inf Lös 20 g/200ml i.v. Durchst f 200 ml<br><br>P0907 - Todesfallkosten	Yes
Mengeneinheit	Unit of measurement	ST, CHF, MIN, (blank)	No
Kosten_Total Rekole	Costs		Yes
Übergeleitete Menge (CO)	Amount transferred (variable of controlling department)		No

Abbreviations: Aufne aufnehmende (receiving); CHF Swiss Francs; CO controlling; Erbr erbringende (executing); Entl entlassende (discharging); Hier Hierarchie (hierarchy); ID identifier; Nr Nummer (number); OE Organisationseinheit (organizational unit); MIN minimum; ST Stück (piece)

S5 Table. Differences and particularities in the structure of the three datasets and resulting challenges

Dataset	Variable	Comments
Clinical dataset	Treatment dates	Sometimes, HEARTBEAT® contained planned future treatment end dates or no end date in case of a currently ongoing treatment. In these cases, we set the treatment end dates to the latest sensible date.
	PROMs	Although prospectively planned for, the PROMs could not always be measured at the exact planned timepoints (BL, 3 months, 6 months, 12 months) at USB. For example, a surgery might have initially started as a diagnostic intervention and turned out to be the treatment start. In that case, the baseline PROM measurement could only be collected through patient interviews after the surgery and after patient consent. Also, other quality of life C30 measurement timepoints recorded in HEARTBEAT® sometimes varied around planned dates, with variation around 1 or 2 months not being uncommon. For this reason, we also allowed PROM measurements up to 15 months after diagnosis (instead of 12 months) for our interim proof of concept analysis, but only if these measurements had officially been declared as the "12-month follow-up" assessment in HEARTBEAT®. In case a PROM measurement and an assigned follow-up assessment month (e.g. 3-month) after diagnosis were available, but no actual PROM assessment date, the date was imputed based on the diagnosis date plus the planned follow-up month.
Service dataset	Service date	A missing "service date" occurred in the originally delivered service dataset with 2,842/757,385~0.4% entries. A missing service date was replaced with an existing receiving date (at the organisational unit), or if also missing with an existing discharging date. There were 1,123/757,385~0.1% entries with neither a service, receiving or discharging date. These data were not retained for our analysis as no time of performance could be assigned at all.<br><br>With a few exceptions, claims with missing service dates also did not contain any information in the service text, service type, or the tariff type. However, although such individual claims were excluded from our reduced datasets, their costs were part of the overall costs per case calculation if other claims in the same case contained adequate date information.
Service and cost dataset	Case number	For the creation of new case numbers, ambulant and inpatient care were distinguished.<br><br>For the FAS population, there were 29 entire cases (for 25 different patients) which were present in the service but with no claims at all in the REKOLE® cost dataset, constituting 29/1948=1.5%. We attributed these findings to data error. The percentage was low.<br><br>Since a further service could be billed within a previous and hence already open case number (=ward) during 12 months after opening, it could happen that services and costs that had occurred slightly later than the interim analysis lock date at the same ward were included in our interim analysis. That was unavoidable when working with costs of entire cases.
Service and cost dataset	Case type	A particular case contained either ambulant, ambulant/day clinic or inpatient services. We assumed ambulant and ambulant/day clinic entries as similar and combined these into an ambulant case type only. Ambulant/day clinic entries were not very common.
Service and cost dataset	Discharging nursing unit, all units	Next to the case number and the case type (ambulant, inpatient), receiving and discharging nursing and specialist units were generally consistent across the service and the cost dataset and stayed the same for all claims (=entries) within the same case.

Dataset	Variable	Comments
		Mismatches were noted in the discharging nursing units of both datasets for 155/1948=8% entire cases in the FAS population. We attributed these findings to data error.<br>Additional note: While in the cost dataset, the executing nursing unit always stayed the same within the same case and corresponded 100% to the discharging nursing unit, the variable with the same name varied in the service dataset (see example in brown colour in **Table S6**).
Service and cost dataset	Service text	Individual linking between the service and cost datasets of claims via the common variable "service text" (within the same case) was not possible. Unclear allocation was primarily caused by multiple entries of the same service description for different time points within the same case, as the cost dataset did not contain an exact service date.<br><br>We also saw that the variable "service text" was not always present with the same entries in both datasets and could sometimes also be blank. We further realised that in the latter cases the controlling variable "amount transferred" was sometimes higher than the costs. The finance department advised that such cases are specific to REKOLE®. Service texts are sometimes blank because they represent in such cases a flat-rate amount for hospital pharmacies.
Service and cost dataset	All variables	A further particularity was the fact that the same claim that had occurred once in the service dataset could be listed and hence billed to several cost types / cost centres in the cost dataset, leading to multiple rows in the cost dataset for one row in the service dataset. As an example, technical service ("TL") and physician service ("AL") are billed separately in the cost dataset.
Cost dataset	Unit of measurement	The finance department reported that the delivered cost variable represented total costs of a service independent of "unit of measurement" (independent if e.g. piece or CHF).

Abbreviations: AL ärztliche Leistung (physician service); BL baseline; CHF Swiss francs; FAS full analysis set; PROM patient reported outcome measure; TARMED Swiss outpatient billing system; TL technische Leistung (technical service); USB University Hospital Basel

S6 Table. Few example entries in the service dataset

EHR_ID	Case_ID	Case_type	Services date	Receiving nursing unit	Receiving specialist unit	Discharging nursing unit	Discharging specialist unit	Executing nursing unit (additionally varies within the same case)	Service text	Service type	Tariff type	DRG, ICD, rec_date, dis_date
1234567	88888881	inpatient	01/01/20020	4800 – emergency department	753 – emergency medical care	4640 – chirurgie 4.1	348 – thoraxic surgery	e.g. 4800 – emergency department, ...	Consultation first 5 mins	tarmed - tarmed-leistung	001 - tariftyp tarmed	...
1234567	88888881	inpatient	01/01/20020	4800 – emergency department	753 – emergency medical care	4640 – chirurgie 4.1	348 – thoraxic surgery	e.g. 4800 – emergency department, ...	00.0026 - consultation for persons over 6 and under 75 years of age with an increased need for treatment, every additional 5 min.	tarmed - tarmed-leistung	001 - tariftyp tarmed	...
1234567	88888881	inpatient	03/01/2020	4800 – emergency department	753 – emergency medical care	4640 – chirurgie 4.1	348 – thoraxic surgery	e.g. 4700 – imc, ...	Consultation first 5 mins	tarmed - tarmed-leistung	001 - tariftyp tarmed	...

Related example entries in the cost dataset (multiple rows in the cost dataset for 1 row in the service dataset is possible)

**Case_ID**	**Merged Case_ID**	**Case_type**	**Receiving nursing unit**	**Receiving specialist unit**	**Discharging nursing unit**	**Discharging specialist unit**	**Executing nursing unit**	**Service text**	**Cost_type**	**Cost_level_5**	**Cost_partner**	**Cost_hier**	**Costs**
88888881	88888881	inpatient	4800 – emergency department	753 – emergency medical care	4640 – chirurgie 4.1	348 – thoraxic surgery	4640 – chirurgie 4.1	Consultation first 5 mins	2500 - notfall	nfze - notfallzentrum	343203.25 - notfallzentrum pfleg	25	5 CHF
88888881	88888881	inpatient	4800 – emergency department	753 – emergency medical care	4640 – chirurgie 4.1	348 – thoraxic surgery	4640 – chirurgie 4.1	Consultation first 5 mins	3100 - ärzteschaften akt. 1 – 5	thch - thoraxchirurgie	303001.31 - thoraxchir ärzte sta	(blank)	10 CHF
88888881	88888881	inpatient	4800 – emergency department	753 – emergency medical care	4640 – chirurgie 4.1	348 – thoraxic surgery	4640 – chirurgie 4.1	00.0026 - + consultation for persons over 6 and under 75 years of age with an increased need for treatment, every additional 5 min.	2500 - notfall	nfze - notfallzentrum	343203.25 - notfallzentrum pfleg	25	30 CHF
88888881	88888881	inpatient	4800 – emergency department	753 – emergency medical care	4640 – chirurgie 4.1	348 – thoraxic surgery	4640 – chirurgie 4.1	00.0026 - + consultation for persons over 6 and under 75 years of age with an increased need for treatment, every additional 5 min.	3150 - ärzteschaften akt. 6 notfall al	nfze - notfallzentrum	343201.31 - notfallz. aerzt stat	(blank)	25 CHF
88888881	88888881	inpatient	4800 – emergency department	753 – emergency medical care	4640 – chirurgie 4.1	348 – thoraxic surgery	4640 – chirurgie 4.1	Consultation first 5 mins	3801 - intermediate care (imc)	imc - intermediate care	341201.38 - imc pflege	38	X CHF
88888881	88888881	inpatient	4800 – emergency department	753 – emergency medical care	4640 – chirurgie 4.1	348 – thoraxic surgery	4640 – chirurgie 4.1	Consultation first 5 mins	3150 - ärzteschaften akt. 6 notfall al	nfze - notfallzentrum	343201.31 - notfallz. aerzt stat	(blank)	X CHF

Abbreviations: CHF Swiss francs; ID identification number

S7 Table. Snippet of derived and grouped service/cost/clinical dataset after step 1 (simplified)

EHR_ID (=Patient identifier)	CASE number	First service date within case number[1]	Last service date within case number[1]	CASE type	Receiving nursing unit	Receiving specialist unit	Discharging nursing unit	Discharging specialist unit	Summed cost per case number (in CHF)[2]	DRG (inpatient only)	ICD (inpatient only)	Patient FU start date (diagnosis – 11 days)[3]	Patient FU end date (=Last FU date)[3]	Other variables from clinical dataset (e.g. surgery 1 date, chemotherapy 1 start date, chemotherapy 1 end date, death, PRO, ... [same dates in all rows related to the same patient]
1111117	88888888	01/01/21	15/01/21	Out-patient	3310 – oncology	3310 - oncology	3310 - oncology	3310 - oncology	10,000	-	-	15/12/20	14/12/21	xx/xx/xxxx
1111117	11111111	06/03/21	14/12/21	Out-patient	2140 - radio-oncology	156 - radio oncology /radiotherapy	2140 - radio-oncology	156 - radio oncology /radiotherapy	750	-	-	15/12/20	14/12/21	xx/xx/xxxx
1111117	22222222	23/05/21	10/06/21	In-patient	3480 – thorax surgery	348 – thorax surgery	3480 – thorax surgery	348 – thorax surgery	100,000	e71a - neoplasms of the respiratory organs with extremely severe complications or rigid bronchoscopy, with moderately or highly complex ..	c34.1 - malignant neoplasm of the upper lobe (- bronchus)	15/12/20	14/12/21	xx/xx/xxxx
1111117	33333333	xx/xx/xx	xx/xx/xx	In-patient	4150 - medicine 7.2	320 - internal medicine	4150 - medicin 7.2	320 – internal medicine	30,000	e71c - neoplasms of the respiratory organs, more than one day of occupancy	c34.1 - malignant neoplasm of the upper lobe (- bronchus)	15/12/20	14/12/21	xx/xx/xxxx
1111117	44444444	xx/xx/xx	xx/xx/xx	In-patient	4180 - medicine 4.1 / fast track	332 - pneumology	4150 - medicine 7.2	320 – internal medicine	15,000	e65c - chronic obstructive pulmonary disease with rigid bronchoscopy, more than one day of occupancy...	j44.19 - chronic obstructive pulmonary disease with acute exacerbation ...	15/12/20	14/12/21	xx/xx/xxxx
......														

[1]from services dataset, [2]from cost dataset, [3]from derived clinical dataset (Table S2)

Abbreviations: CHF Swiss francs; DRG diagnosis related group; EHR electronic health record; FU follow-up; ICD International Statistical Classification of Diseases and Related Health Problems; ID identification number

S8 Table. Medication search list for chemotherapy, immunotherapy and targeted therapy

Search strings for chemotherapy*	Search strings for immunotherapy*	Search strings for targeted therapy*	
Abraxan	Atezolizumab	Adagrasib	1
Adriblastin	Avastin	Afatinib	2
Alimta	Bevacizumab	Alecensa	3
Bendamustin(e)	Cemiplimab-rwlc	Alectinib	4
Caelyx	Cyramza	Alunbrig	5
Campto	Durvalumab	Brigatinib	6
Carboplatin	Imfinzi	Cabometyx	7
Cav**	IMJUDO	Cabozantinib	8
Cisplatin	Ipilimumab	Capmatinib	9
Cyclophosphamid(e)	Keytruda	Ceritinib	10
Docetaxel	LIBTAYO	Crizotinib	11
Doxorubicin	Mvasi	Dabrafenib	12
Endoxan	Nivolumab	Dacomitinib	13
Etopophos	Opdivo	Enhertu	14
Etoposid	Oyavas	Entrectinib	15
Gemcitabin	Pembrolizumab	Erlotinib	16
Gemcitabine	Ramucirumab	Exkivity	17
Hycamtin	Tecentriq	Fam-trastuzumab deruxtecan-nxki	18
Irinotecan	Tremelimumab-actl	Gavreto	19
Lurbinectedin	Yervoy	Gefitinib	20
Navelbin(e)	Zirabev	Giotrif	21
Oncovin Liquid		Iressa	22
Paclitaxel		Kadcyla	23
Paraplatin		KRAZATI	24
Pemetrexed		Larotrectinib	25
Ribomustin		Lorlatinib	26
Taxol		Lorviqua	27
Taxotere		Lumykras	28
Temodal		Mekinist	29
Temozolomid(e)		Mobocertinib	30
Topotecan		Osimertinib	31
Vepesid		Pralsetinib	32
Vincristin(e)		Retsevmo	33
Vinorelbin(e)		Rozlytrek	34
Zepzelca		Selpercatinib	35
		Sotorasib	36
		Tabrecta	37
		Tafinlar	38
		Tagrisso	39
		Tarceva	40
		Tepmetko	41
		Tepotinib	42
		Trametinib	43
		Trastuzumab emtansine	44
		Vemurafenib	45
		Vitrakvi	46
		Vizimpro	47
		Xalkori	48
		Zelboraf	49
		Zykadia	50

*Capitalisation had been standardised before the search

** Search for entire individual word, not only for string

S9 Table. Search procedure to assign treatments to case numbers (second step of merge strategy)

Order	Search string for	Overview	R code snippets:
1	Surgery	- Hospital inpatient setting only &<br><br>- Surgery date within case start and end dates &<br><br>- Relevant strings in the service text [gve02c|gve05a|gve05b|kte02c|kte05a|kte05b|zve05b] OR the service_type must contain one of the three options: "opsl – operationsleistung, drg – ltyp, "implantate" &<br><br>- Receiving, executing, or discharging nursing or specialist organisational unit should have been "Thoraxchirurgie", OR the executing nursing organisational unit should have been "operation room" ["2003 – operationssäle"].<br><br>- Only if a patient had an inpatient surgery, further ambulant service claims were assigned to surgery in case both executing and discharging nursing units were thoraxchirurgie in the service dataset, or the executing (=discharging) nursing unit was thoraxchirurgie in the REKOLE® dataset	CASE_type=="inpatient" &<br><br>CASE_starting_date <= surgery_date <= CASE_end_date &<br><br>(grepl("gve02c|gve05a|gve05b|kte02c|kte05a|kte05b|zve05b", service_text) | <br>(grepl("opsl - operationsleistung", service_type) | grepl("drg - ltyp",service_type) | grepl("implantate",service_type))) &<br><br>((rowSums(across(ends_with("_unit"), ~str_detect(., "348 - thoraxchirurgie|353")) > 0))| str_detect(exe_nurs_unit, "2003 - operationssäle")).<br><br>((exe_nurs_unit=="3480 - thoraxchirurgie" & dis_nurs_unit=="3480 - thoraxchirurgie") | grepl("thoraxchirurgie",exe_nurs_unit_rek))
2	Radiotherapy	Only for patients with identified radiotherapy based on heartbeat information:<br><br>In cost dataset:<br>Search for keywords related to nuclear medicine and radioonkologie in the variable "cost type". Retain identified case numbers.<br><br>Search in service dataset:<br>Search for strings related to radiotherapy in service text (e.g. "high voltage radiation therapy", "acceleration therapy" but exclude German expressions for "without radiotherapy" or" without radiation therapy", "X-ray", "colon", "lobectomy").<br>Retain identified case numbers.<br><br>Reduce service dataset to identified cases from both searches and subset to claims within window (=11) days before radiotherapy start and window days after radiotherapy enddate (based on heartbeat information). Retain list of final case numbers for radiotherapy.	filter(grepl("R", path_seq))<br><br>grepl("2800 - 2800 - nuklearmedizin und radioonkologie al|2810 - nuklearmedizin und radioonkologie tl", cost_type)<br><br>filter((grepl("hochvoltstrahlentherapie | hochvolt | strahlentherapie| strahl | beschleunigertherapie| bestrahl", service_text) &<br>!grepl("ohne strahlentherapie| röntgen | dickdarm | lobektomie", service_text))<br><br>s_date>=startdate_1 - window &<br>s_date<=stopdate_last + window

Order	Search string for	Overview	R code snippets:
3	Systemic chemotherapy, immunotherapy or targeted therapy	Search for medication names in the variable "service_text" in both the cost and the service dataset (**Table S8).** Retain case number if hit in at least one of the two datasets. Keep both costs of the entire case and costs of identified drugs within the case.<br><br>Optional restriction for faster programming runtime to "cost_type=4000 - arzneimittel" in service dataset and to "service_type=="med - medikamente ohne tagesdosis" or service_type= "drg - ltyp 1 drg" & tarif_type= "011 – zusatzentgelt" in cost dataset.<br><br>No check if medications were given during the treatment interval recorded in heartbeat or if case had already been assigned (but could easily be added)<br>If multiple assignments of different treatments to the same case number (e.g. chemo- and immunotherapy, or chemo- and radiotherapy), costs were split based on retained drug and radiation costs previously identified. Remaining costs were split equally.	
4	Death in hospital	If patient died at hospital (death location=hospital) &<br>Case number still unassigned &<br>Death happened within case number start and end date	
6	Diagnosis before first treatment	Based on the diagnosis and the first treatment date (Heartbeat system), we allocated all open case numbers to diagnosis in case they occurred within the time interval window (11 days) days before the diagnosis date and 1 day before the first treatment date.<br>The 1 patient without any treatment had all cases assigned to diagnosis.	
7	Remaining open case numbers	We assigned all remaining case numbers to "other" and assumed that these consisted of any comorbidity treatment, FU treatment without billed drugs, inpatient complications or progression not already captured in previous case numbers of the related treatment.	

Abbreviations: drg diagnosis related group; dis_nurs_unit discharging nursing unit; exe_nurs_unit executing nursing unit; FU follow-up; opsl operation service; R radiotherapy; rek REKOLE®; seq sequence

S10 Table. Snippet of derived and grouped service/cost/clinical dataset AFTER STEP 2

EHR_ID (=Patient identifier)	CASE number	First date within case number[1]	Last date within case number[1]	CASE type	Receiving nursing unit	Receiving specialist unit	Discharging nursing unit	Discharging specialist unit	Summed cost per case number (in CHF)[2]	DRG (inpatient only)	ICD (inpatient only)	Patient FU start date (diagnosis – 11 days)[3]	Patient FU end date (=Last FU date)[3]	Other variables from clinical dataset (e.g. surgery 1 date, chemotherapy 1 start date, chemotherapy 1 end date,...[3]	**Assigned treatment per case number after step 2**
1111117	88888888	01/01/21	15/01/21	Out-patient	3310 – oncology	3310 - oncology	3310 - oncology	3310 - oncology	10,000	-	-	15/12/20	14/12/21	xx/xx/xxxx	**Diagnosis**
1111117	11111111	06/03/21	14/12/21	Out-patient	2140 - radio-oncology	156 - radio oncology /Radiotherapy	2140 - radio-oncology	156 - radio oncology /Radiotherapy	750	-	-	15/12/20	14/12/21	xx/xx/xxxx	**Radio-therapy**
1111117	22222222	23/05/21	10/06/21	In-patient	3480 – thorax surgery	348 – thorax surgery	3480 – thorax surgery	348 – thorax surgery	100,000	xxxxxxx	xxxxxx	15/12/20	14/12/21	xx/xx/xxxx	**Surgery**
1111117	33333333	xx/xx/xx	xx/xx/xx	In-patient	4150 - medicine 7.2	320 - internal medicine	4150 - medicin 7.2	320 – internal medicine	30,000	xxxxxxx	xxxxxx	15/12/20	14/12/21	xx/xx/xxxx	**Immuno-therapy**
1111117	44444444	xx/xx/xx	xx/xx/xx	In-patient	4180 - medicine 4.1 / fast track	332 - pneumology	4150 - medicine 7.2	320 – internal medicine	15,000	xxxxxxx	xxxxxx	15/12/20	14/12/21	xx/xx/xxxx	**Other**

[1]from service dataset, [2]from cost dataset, [3]from derived clinical dataset (Table A2)

Abbreviations: CHF Swiss francs; DRG Diagnosis related group; EHR electronic health record; FU follow-up; ICD International Statistical Classification of Diseases and Related Health Problems; ID identification number

S11 Table. Distribution of included lung patients by stage and histology (full analysis set, N=208)

Table 1. Distribution of patients by histology and stage: FAS

	Histology							
	Adenocarcinoma	Squamous cell carcinoma	Large cell neuroendocrine carcinoma	Small cell lung carcinoma	Lung Carcinoma NOS	Thoracic tumours	Others	**Total**
**LC Stage**								
I	67 (32%)	11 (5.3%)	1 (0.5%)	2 (1.0%)	2 (1.0%)	2 (1.0%)	4 (1.9%)	89 (43%)
II	10 (4.8%)	4 (1.9%)	1 (0.5%)	2 (1.0%)	0 (0%)	2 (1.0%)	0 (0%)	19 (9.1%)
III	17 (8.2%)	15 (7.2%)	1 (0.5%)	6 (2.9%)	0 (0%)	1 (0.5%)	0 (0%)	40 (19%)
IV	36 (17%)	7 (3.4%)	1 (0.5%)	13 (6.3%)	1 (0.5%)	1 (0.5%)	1 (0.5%)	60 (29%)
**Total**	130 (63%)	37 (18%)	4 (1.9%)	23 (11%)	3 (1.4%)	6 (2.9%)	5 (2.4%)	208 (100%)

Abbreviation: LC lung cancer; NOS not otherwise specified

S12 Table. Baseline patient characteristics; number and type of comorbidities

Variable	N = 208[1]	Variable	N = 208[1]
<u>**Age at diagnosis**</u>		<u>**Comorbidities**</u>	
Mean (SD)	70 (11.1)	**Heart disease**	44 / 182 (24%)
Median (IQR)	71 (62, 78)	Missing	26
Range	21, 92	**High blood pressure**	70 / 182 (38%)
		Missing	26
**Female Gender**	81 / 208 (39%)	**Leg pain**	15 / 182 (8.2%)
		Missing	26
<u>**Highest education**</u>		**Lung disease[2]**	39 / 182 (21%)
Primary school	22 / 183 (12%)	Missing	26
Secondary school	108 / 183 (59%)	**Diabetes**	16 / 182 (8.8%)
Higher education (including university degree)	53 / 183 (29%)	Missing	26
Missing	25	**Kidney disease**	11 / 182 (6.0%)
		Missing	26
<u>**Number of comorbidities**</u>		**Liver disease**	1 / 182 (0.5%)
0	50 / 182 (27%)	Missing	26
1	62 / 182 (34%)	**Stroke**	13 / 182 (7.1%)
2	33 / 182 (18%)	Missing	26
3	22 / 182 (12%)	**Nervous system**	4 / 182 (2.2%)
4	7 / 182 (3.8%)	Missing	26
5	5 / 182 (2.7%)	**Other cancer**	27 / 182 (15%)
6	2 / 182 (1.1%)	Missing	26
7	1 / 182 (0.5%)	**Depression**	10 / 182 (5.5%)
Missing	26	Missing	26
		**Arthritis**	27 / 182 (15%)
		Missing	26

[1] n / N (%), [2] for example angina, heart attack, heart failure

Abbreviation: N number of patients in full analysis set population

S13 Table. Summary of first year hospital costs of lung cancer treatment per cost type (full analysis set, N=208)

Case type	N	Total first-year hospital costs (in CHF)	Percent	Median first year costs per patient (in CHF)	Mean (SD) first year costs per patient (in CHF)	[Min; Max] first year costs per patient (in CHF)
Outpatient	203	5.43 Mio	28%	13,049	26,744 (30,949)	[371; 168,104]
Inpatient	208	14.04 Mio	72%	49,863	67,519 (56,102)	[2,939; 433,104]
Total costs		19.47 Mio		77,834	93,621 (64,548)	[16,944; 473,160]

Abbreviations: CHF Swiss francs; N sample size; SD standard deviation

S14 Table. Example correlation results (Spearman, Partial Correlation (SEM))

Histology	Stage	n	Spearman	Partial correlation		Strength and direction of correlation*
			Correlation coefficient	Covariance estimate	Std.all (total_cost_patient ~~ cfb_utility)	
Adenocarcinoma	I	54	-0.00482	80.46	0.007	None
	II	10	0.338	Matrix not positive definite		None to medium positive
	III	15	-0.0324	240.64	0.022	None
	IV	32	-0.224	-4807.84	-0.307	Low to medium negative
Squamous cell carcinoma	I	10	0.569	12411.19	0.509	High positive
	II	4	0.0665	-104455.26	-0.132	None to low negative
	III	15	-0.203	-2727.96	-0.187	Low negative
	IV	7	0.238	4222.98	0.821	Low to very high positive

*** These interim results should be interpreted with great caution due to the small sample sizes and the presence of outliers in both cost and utility values. Also, the absence of standard errors and p-values indicated some limitations in the model's reliability, but the standardised results can still offer valuable insights into the relationships between the variables.**

Abbreviation: cfb change from baseline; n subgroup sample size; SEM structural equation model; Std.all standardised coefficient

S15 Table. Executing nursing units (in German) assigned to category “other”

1	1410 - ernährungsberatung
2	1500 - sozialdienst
3	2050 - anästhesie allgemein/op
4	2051 - schmerzmedizin usb
5	2052 - anästhesie pas
6	2130 - nuklearmedizin
7	2140 - radio-onkologie
8	2141 - molekul. diagn. hämatologie
9	2145 - tb radio-onkologie
10	2209 - leitung laboratorien
11	2210 - hämatologielabor
12	2225 - poct chemie
13	2229 - chemisches labor
14	2249 - bakteriologielabor
15	2250 - hämostaselabor
16	2251 - immunologie
17	2310 - physiotherapie medizin/ufk
18	2320 - physiotherapie chirurgie
19	2340 - lm virologie
20	2390 - immunphänotypisierung
21	2600 - konven. röntgendiagn.
22	2610 - computertomographie
23	2620 - magnet-resonanz-tomographie
24	2630 - ultraschall diagn.radiol.
25	2640 - angioradiologie
26	2650 - diagn. radiologie
27	2670 - kardiologie ambulant
28	2680 - kardio überwachung
29	2690 - kardiovaskuläre prävention
30	3006 - pneumologie bethesda
31	3020 - angiologie bethesda
32	3050 - clarunis gastro bethesda
33	3060 - endoskopie pneumo
34	3230 - angiologie
35	3240 - diabetologie
36	3250 - clarunis gastroenterologie
37	3260 - hämatologie
38	3300 - nephrologie/dialyse
39	3301 - nephrologie ambulatorium
40	3310 - onkologie
41	3320 - pneumologie/lungenfunktion
42	3330 - klin.pharmakologie
43	3340 - psychosomatik
44	3350 - clarunis stomaberatung
45	3360 - klinische infektiologie
46	3381 - neuro eeg
47	3383 - neuro emg
48	3385 - neuro cerebrov.
49	3420 - spinale chirurgie
50	3480 - thoraxchirurgie
51	3511 - clarunis viszeralchirurgie
52	3530 - neurochirurgie
53	3541 - mund-,kiefer-&gesichtschir.
54	3542 - plastische chirurgie
55	3560 - gefässchir. & organtranspl.
56	3580 - urologie inklusive polikl.
57	3600 - hno kopf/hals/tumorzentrum
58	3614 - ambulanz gellertstrasse 144
59	3641 - hno audiologie
60	3670 - ak diagnostik
61	3690 - ak orthoptik
62	3730 - dermatologische klinik
63	3780 - genetik ärzte
64	3860 - biopsie pathologie
65	3880 - zytopathologie
66	3900 - molekular pathologie
67	3951 - pathologie administration
68	4050 - stroke unit
69	4070 - privat-u.spezialsprechstd.
70	4090 - stroke center administration
71	4100 - medizin 7.1
72	4120 - isolierstation
73	4122 - zellersatz-ambulatorium
74	4130 - medizin 6.2
75	4140 - medizin 5.1
76	4150 - medizin 7.2
77	4161 - med.kurzzeitklinik
78	4180 - medizin 4.1 / fast track
79	4200 - clarunis gastro praxis fps
80	4310 - de bettenstation
81	4410 - neurologie 4.2
82	4610 - chirurgie 6.2
83	4620 - chirurgie 3.1
84	4640 - chirurgie 4.1
85	4650 - chirurgie 5.1
86	4660 - chirurgie 6.1
87	4670 - chirurgie 7.1
88	4710 - hno bettenstation
89	4720 - tagesklinik chirurgie
90	4750 - chirurgie 5.2
91	4770 - chirurgie 7.2
92	4782 - urologie uafp
93	4790 - ortho bethesda
94	4800 - notfallzentrum
95	4920 - fk gyn. bettenstation
96	5730 - medizin 8.1
97	7340 - brustchirurgie
98	7410 - hno poliklinik
99	7411 - hno phoniatrie
100	7420 - triage- und testcenter
101	7500 - amb. plattform innere medizin
102	7510 - chirurgische poliklinik
103	7540 - de poliklinik
104	7550 - de allerg. poliklinik
105	7560 - neurologische poliklinik
106	7595 - fk mamma-sprechstunde
107	7650 - ak poliklinik
108	7654 - ak glaukom ss
109	7656 - ak retina ss
110	7657 - ak klin. pathologie ss
111	7670 - de wundambulatorium
112	7682 - fk amb. gyn. tumorzentrum
113	#N/A

References

[1] M.E. Charlson, P. Pompei, K.L. Ales, C.R. MacKenzie, A new method of classifying prognostic comorbidity in longitudinal studies: development and validation, Journal of chronic diseases 40(5) (1987) 373-383.
[2] B. Colinet, W. Jacot, D. Bertrand, S. Lacombe, M. Bozonnat, J. Daures, J. Pujol, A new simplified comorbidity score as a prognostic factor in non-small-cell lung cancer patients: description and comparison with the Charlson's index, British journal of cancer 93(10) (2005) 1098-1105.
